# Blood mercury, lead, cadmium, manganese and selenium levels in pregnant women and their determinants: the Japan Environment and Children’s Study (JECS)

**DOI:** 10.1038/s41370-019-0139-0

**Published:** 2019-04-18

**Authors:** Shoji F. Nakayama, Miyuki Iwai-Shimada, Tomoko Oguri, Tomohiko Isobe, Ayano Takeuchi, Yayoi Kobayashi, Takehiro Michikawa, Shin Yamazaki, Hiroshi Nitta, Toshihiro Kawamoto, the Japan Environment and Children’s Study Group, Hirohisa Saito, Reiko Kishi, Nobuo Yaegashi, Koichi Hashimoto, Chisato Mori, Shuichi Ito, Zentaro Yamagata, Hidekuni Inadera, Michihiro Kamijima, Takeo Nakayama, Hiroyasu Iso, Masayuki Shima, Yasuaki Hirooka, Narufumi Suganuma, Koichi Kusuhara, Takahiko Katoh

**Affiliations:** 1 Japan Environment and Children’s Study Programme Office, National Institute for Environmental Studies, 16-2 Onogawa, Tsukuba Ibaraki, 305-8506 Japan; 2 Research Institute of Science for Safety and Sustainability, National Institute of Advanced Industrial Science and Technology (AIST), 16-1 Onogawa, Tsukuba Ibaraki, 305-8569 Japan; 3 Department of Preventive Medicine and Public Health, Keio University, 35 Shinanomachi, Shinjukuku Tokyo, 106-8582 Japan; 4 Department of Environmental and Occupational Health, School of Medicine, Toho University, 5-21-16 Omori-nishi, Otaku Tokyo, 143-8540 Japan; 5 National Centre for Child Health and Development, Tokyo, Japan; 6 Hokkaido Regional Center for JECS, Hokkaido University, Sapporo, Japan; 7 Tohoku University, Sendai, Japan; 8 Fukushima Medical University, Fukushima, Japan; 9 Chiba University, Chiba, Japan; 10 Yokohama City University, Yokohama, Japan; 11 University of Yamanashi, Chuo, Japan; 12 University of Toyama, Toyama, Japan; 13 Nagoya City University, Nagoya, Japan; 14 Kyoto University, Kyoto, Japan; 15 Osaka University, Suita, Japan; 16 Hyogo College of Medicine, Nishinomiya, Japan; 17 Tottori University, Yonago, Japan; 18 Kochi University, Nankoku, Japan; 19 University of Occupational and Environmental Health, Kitakyushu, Japan; 20 Kumamoto University, Kumamoto, Japan

**Keywords:** Japan Environment and Children’s Study (JECS), Birth cohort, Mercury, Lead, Cadmium, Manganese

## Abstract

The Japan Environment and Children’s Study (JECS) is a birth-cohort study of 100,000 mother–child dyads that aims to investigate the effect of the environment on child health and development. Mercury (Hg), lead (Pb), cadmium (Cd), manganese (Mn) and selenium (Se) are considered to be important co-exposures when examining the effect of other chemical substances on child development. The levels of these elements in the blood of 20,000 randomly selected mid/late-term pregnant women from the whole JECS cohort were analysed using inductively coupled plasma-mass spectrometry. The median concentrations (interquartile ranges) for Pb, Hg, Cd, Mn and Se were 0.63 (0.51–0.78) µg dl^−1^, 3.83 (2.70–5.43) µg l^−1^, 0.70 (0.52–0.95) µg l^−1^, 16.1 (13.2–19.6) µg l^−1^ and 178 (165–192) µg l^−1^, respectively. Hg and Se correlated positively with each other (Spearman’s *ρ* = 0.287), as did Pb and Cd (*ρ* = 0.239) and Cd and Mn (*ρ* = 0.267). The blood Pb levels decreased by 5–10-fold over the past 25 years. The main predictors of the blood levels of each element were fish consumption for Hg, maternal age and non-alcoholic beverage consumption for Pb, maternal age and smoking for Cd, gestational age at sampling for Mn and serum protein levels for Se. These results revealed the historical trends and current predictors of the blood levels of these elements in pregnant Japanese women.

## Introduction

The Ministry of the Environment of Japan has been conducting a large-scale birth-cohort study since 2011 to examine the effect of various environmental factors on child health and development. This study is called the Japan Environment and Children’s Study (JECS) and it involves more than 100,000 mother–child dyads [1, 2]. It assesses the effect of the environment, especially exposure to chemical substances, on the rate of reproduction/pregnancy complications, congenital anomalies, developmental disorders and immune/metabolic system dysfunction. Environmental exposure is assessed by using self-administered questionnaires, by measuring the ambient environment, by conducting numerical modelling and, in particular, by collecting and analysing biological samples such as blood, urine, hair and breast milk. Participant recruitment ended in March 2014 [2]. The study registered a total of 103,099 pregnancies that resulted in 100,778 deliveries, of which 100,148 were live births. All babies were born by 2015. Biospecimens were collected during pregnancy, at birth and at the first month check-up. After recruitment and sample collection was completed in 2015, we started analysing the biological samples for chemical contaminants.

Heavy metals such as lead (Pb), cadmium (Cd) and mercury (Hg) have adverse effects on child health and development [3–5]. Manganese (Mn) is one of the trace metals that are essential for human development and metabolism; it also has antioxidant properties. However, it is neurotoxic when humans are exposed to it in early childhood [6–9]. Selenium (Se) is also important for human health but has a narrow ‘safe’ level and is toxic at high levels [10]. Se interacts with Hg and may modify the toxicological consequences of Hg exposure [11]. These findings suggest that these elements are important co-exposures when seeking to evaluate the effect of other chemical contaminants on child health. To address this issue, we aimed to analyse the concentrations of these metallic elements in the maternal blood samples of all JECS participants.

In 2015, 20,000 maternal whole blood samples that were collected in mid/late-term pregnancy were randomly selected from all available samples of the same kind and the concentrations of Hg, Pb, Cd, Mn and Se were measured. The data were finalised and distributed to JECS researchers in April 2017. The present study is based on these data and had three purposes. First, it aimed to provide JECS with basic data on the levels of the five metallic element levels: these data can then be used as co-exposure variables when analysing associations between the environment and child health and development. Second, it aimed to illustrate the current exposure status of pregnant Japanese women in general: this is possible because the study recruits were selected so that they closely represent the population of pregnant women in Japan [2]. Third, the study aimed to identify factors that predict the Hg, Pb, Cd, Mn and Se levels in the blood of pregnant Japanese women to determine a way to mitigate unnecessary exposure.

## Materials and methods

### Study participants

The JECS study design has been published elsewhere [1]. Briefly, JECS is funded by the Ministry of the Environment of Japan and operated by the Programme Office (National Institute for Environmental Studies) in co-ordination with the Medical Support Centre (National Centre for Child Health and Development) and 15 Regional Centres (Hokkaido, Miyagi, Fukushima, Chiba, Kanagawa, Koshin, Toyama, Aichi, Kyoto, Osaka, Hyogo, Tottori, Kochi, Fukuoka and South Kyushu/Okinawa). Each Regional Centre determined its own study areas (which consisted of one or more local municipalities) and was responsible for recruiting women in early pregnancy. Between January 2011 and March 2014, we registered 103,099 pregnant women [2]. For the present study, 20,000 participants were randomly selected from all JECS participating mothers who provided blood samples in mid/late-term pregnancy.

The Ministry of the Environment’s Institutional Review Board on Epidemiological Studies and the ethics committees of all participating institutions approved the JECS protocol. The study was conducted according to the tenets of the Declaration of Helsinki and its revisions. Written informed consent was obtained from all mothers who participated in the study.

### Sample collection

Maternal whole blood samples were used to analyse the associations between foetal exposure to five metallic elements and children’s health and development. While the best surrogate for foetal exposure is cord blood, maternal blood was selected because we thought it should be used for subsequent development of reference standards based on the study results. Blood samples (33 ml) were collected by medical staff when the participants visited co-operating health care providers [1]. Phlebotomy devices, including needles and vacutainers, were provided by the Programme Office through its contract laboratory. Of the 33 ml, 9 ml was collected into a vacutainer with coagulant. The remaining blood was placed into four containers (one 3-ml tube and three 7-ml tubes) with sodium ethylenediaminetetraacetic acid (EDTA). The collected samples were then transferred to the contract laboratory within 48 h via land or air transportation.

The 9 ml sample was allowed to separate into serum and the coagulated blood cells. Of the ~4 ml of serum that was obtained from each sample, 2 ml was used for clinical chemistry. The remaining 2 ml was aliquoted into two 2-ml Data Matrix code-labelled cryogenic biobanking tubes (Greiner Bio-One International GmbH, Kremsmünster, Austria). The 3-ml whole blood sample was aliquoted into two 2-ml cryogenic biobanking tubes. The three 7-ml samples were centrifuged to produce plasma samples. Each sample was then aliquoted into two 5-ml cryogenic biobanking tubes (Greiner Bio-One International GmbH).

All vacutainers and cryogenic tubes were tested for potential contamination with the target chemicals: contamination was not evident. Moreover, travel blanks, namely, artificial plasma, were created and aliquoted at some of the study sites according to the same process used during phlebotomy, after which they were transported to and handled and stored in the contract laboratory: analysis of these travel blanks revealed no obvious contamination from any of these procedures. All blood samples were stored at a central biorepository facility at −80 °C. In the present study, we used one of the 2 ml whole blood aliquots obtained from mothers during the second or third trimester.

#### Chemicals and reagents

All reagents were of high-quality grade unless specified otherwise. Water was brought to ≥18 MΩ cm by using a Milli-Q Gradient A-10 system (Merck Millipore, Bedford, MA). Ultrapure-grade nitric acids (1.38 g cm^−3^) and l-cysteine hydrochloride monohydrate (98.0% purity) were purchased from Kanto Chemical Co., Inc. (Tokyo, Japan). Polyoxyethylene (10) octylphenyl ether (POE), practical grade butan-1-ol and 25% w/v super special grade tetramethylammonium hydroxide (TMAH) were purchased from Wako Pure Chemical Industries (Osaka, Japan). EDTA was obtained from Sigma-Aldrich (St Louis, MO, USA). The reference standard solutions (1000 mg l^−1^) of Hg, Cd, Se and indium (In) were purchased from Kanto Chemical Co., Inc. The reference standard solutions (1000 mg l^−1^) of Pb, Mn, yttrium (Y), thallium (Tl) and molybdenum (Mo) were obtained from Wako Pure Chemical Industries.

All glassware, sample tubes and containers were soaked in 10% v/v nitric acid for at least 7 days and rinsed three times with Milli-Q water prior to use.

#### Sample preparation

All solutions were prepared via a gravimetric procedure. Blood samples were brought to room temperature and vortex-mixed prior to preparing aliquots. Quality control (QC), blank water and blood samples (200 µl) were diluted 1:19 (v/v) with the dilution solution, which consisted of 2% (v/v) butan-1-ol, 0.1% TMAH, 0.5 g l^−1^ POE and 0.5 g l^−1^ EDTA, and vortex-mixed prior to inductively coupled plasma-mass spectrometry (ICP-MS) analysis. The calibration range is shown in Table S1. All samples that were outside the calibration range were re-analysed following further dilution.

#### Instrument analysis and calculations

ICP-MS measurements were performed by using an Agilent 7700 ICP-MS (Agilent Technologies, Tokyo, Japan). The typical routine operating conditions and data acquisition settings are documented in Table S2.

The method detection limits (MDLs) for each element were calculated based on Currie’s method [12] using the following equation:

$${\mathrm{MDL}} = t_{\left( {n - 1,0.05} \right)} \times 2 \times s$$

Where *t*_(*n*−1, 0.05)_ represents the Student’s *t* value under an *α* level of 0.05 with *n*−1 degrees of freedom, and *s* represents the standard deviation of blank measurements in *n* replicates (*n* ≥ 7).

Repeatability and intermediate precision were determined on the basis of ISO 5725:1994 and 27148:2010. Briefly, repeatability was determined by analysing the reference standards and pooled QC samples by a single operator using a single machine within a single day. Intermediate precision was derived from the continuous analysis of Seronorm QC samples by multiple personnel using multiple machines for multiple days.

#### Quality control

Seronorm™ Trace Element Whole Blood L-1 (REF 210105, Lot. 1003191, Sero AS, Billingstad, Norway) was used as a QC sample. Blood samples donated by the Japanese Red Cross were used as a pooled QC sample. The QC sample was treated according to the same procedure as whole blood samples and analysed twice in each analytical sequence. The Shewhart control chart ($\bar X$-*R*_m_ chart) was used for QC of day-to-day analysis in accordance with ISO 7870. The ICP-MS method is shown in Table S1. Seven-point calibration curves had coefficients of determination (*R*^2^) higher than 0.9999. The MDLs for Hg, Pb, Cd, Mn and Se were 0.0490, 0.129, 0.0234, 0.522 and 0.837 ng g^−1^, respectively. Repeatability and intermediate precision were 1.6 and 2.5% for Hg, respectively; 0.82 and 1.2% for Pb, respectively; 1.7 and 3.5% for Cd, respectively; 3.4 and 1.4% for Mn, respectively; and 1.4 and 0.89% for Se, respectively (all as relative standard deviation).

**Fig. 1:**
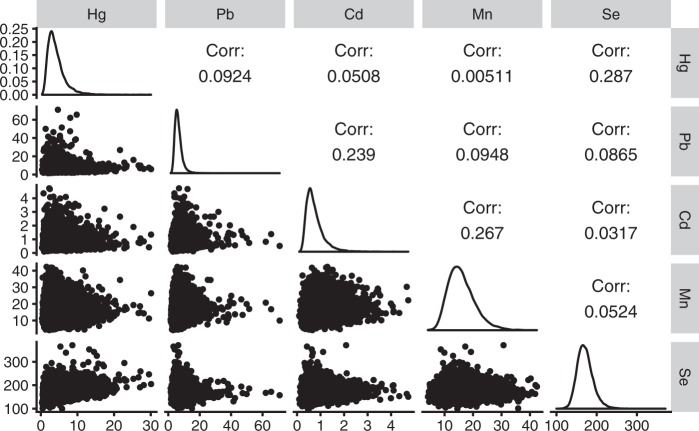
Graphical summary of the distributions of and the correlations between the mercury (Hg), lead (Pb), cadmium (Cd), manganese (Mn) and selenium (Se) concentrations in the maternal blood. The upper matrix shows the Spearman’s rank correlation coefficients. The diagonal cells illustrate the distribution of each element concentration. The lower matrix presents scatter plots with axis units in µg l^−1^

Of the 20,000 blood samples, two were excluded due to the withdrawal of the participant from the study before analysis. The concentrations of the metallic elements in the remaining 19,998 samples were measured in 468 batches. Thereafter, 1000 samples were randomly selected from the 19,998 samples and re-analysed in a different laboratory. Of the 468 batches, 42 batches (1999 samples) were rejected because the data obtained from the re-analysis did not meet the criteria (i.e., that the metallic element concentrations measured by the second laboratory should be within 30% agreement of the measurements obtained in the first laboratory). After the ICP-MS analyses but before data confirmation, a participant withdrew from the study. Another participant was excluded because the collection date of her sample had not been recorded. Thus, in total, 17,997 measurement results were used in this study (Fig. 1). The basic characteristics of the 20,000 and 17,997 subjects did not differ (data not shown).

### Data analysis

A gravimetric procedure that yielded data in gravimetric units (ng g^−1^) was used to precisely aliquot the samples. For comparison with reference values and previous study reports, we converted our measurements in gravimetric units to volumetric concentrations using 1.0506 as a typical specific gravity of whole blood at 37 °C [13]. Statistics in the original units (ng g^−1^) are shown in Table S3. The maternal whole blood Hg, Pb and Cd concentrations were log-transformed (base 10) for statistical analysis because log-normal distribution fitted those elements better. Mn and Se concentrations were used without transformation. The relationships between the blood concentrations of each element and maternal characteristics were examined by using multiple regression analysis. For this, the concentration of each element was treated as the dependent variable while the maternal characteristics served as the independent variables. The maternal characteristics included gestational week at sampling (weeks), age at delivery (years), gestational weight gain (three categories), marital status (two categories), education (three categories), household income (two categories), any smoking during pregnancy (yes/no), any passive smoking during pregnancy (yes/no), any alcohol consumption during pregnancy (yes/no), parity (primipara or not), multiple pregnancy (yes/no), study area (15 Regional Centres) and occupational exposure to hazardous materials in mid/late-term pregnancy (yes/no). Daily food consumption data were categorised into quartiles, except for sugar/sweets and nuts (taken during pregnancy or not). Water consumption (tap/well and bottled) was analysed as frequency data (times per week). These data were obtained by a self-administered questionnaire, a food frequency questionnaire [14] and medical record transcriptions. Maternal characteristics were included in the multiple regression models if they associated with the dependent variable in the bivariate analysis (|*ρ*| > 0.1 by Spearman’s correlation analysis for continuous variables or *p* ≤ 0.0001 by Kruskal–Wallis tests followed by Steel’s multiple comparison Wilcoxon tests for categorical variables; Table S4–6). The food groups were in accordance with a French study [15]. In the final models, the same variables were used for all elements, thus allowing the relationships between all possible predictors and the five elements to be assessed. In compliance with a statement by the American Statistical Association [16], standardised partial regression coefficients (*β*) were presented to directly identify possible predictors of the target element levels in blood. All statistical analyses were conducted using the R version 3.4.0 [17] and JMP 13.0.0 software (SAS Institute Japan Ltd., Tokyo, Japan).

## Results

### Concentrations of Hg, Pb, Cd, Mn and Se in maternal whole blood

The characteristics of the study subjects are shown in Table 1. Hg, Pb, Cd, Mn and Se were detected in all samples (Table 2). The median concentrations (interquartile ranges [IQRs]) of Hg, Pb, Cd, Mn and Se were 3.83 (2.70–5.43) µg l^−1^, 0.63 (0.51–0.78) µg dl^−1^, 0.70 (0.52–0.95) µg l^−1^, 16.1 (13.2–19.6) µg l^−1^ and 178 (165–192) µg l^−1^, respectively. The geometric mean concentrations (95% confidence intervals) of Hg, Pb, Cd, Mn, and Se were 3.83 (3.80–3.86) µg l^−1^, 0.64 (0.63–0.64) µg dl^−1^, 0.71 (0.71–0.72) µg l^−1^, 16.1 (16.0–16.2) and 179 (178–179) µg l^−1^, respectively. The maximum concentrations of Hg, Pb, Cd, Mn and Se were 30.6 µg l^−1^, 7.45 µg dl^−1^, 4.97 µg l^−1^, 44.5 µg l^−1^ and 390 µg l^−1^, respectively.

**Table 1:** Characteristics of the study subjects

	*n*	Unit	Mean	Standard deviation	25th percentile	Median	75th percentile
Gestational week at sampling	17,884	week	27.1	3.2	25.0	27.0	29.0
Age at delivery	17,932	years	31.2	5.0	28.0	31.0	35.0
Body weight before pregnancy	17,983	kg	53.2	8.9	47.2	52.0	57.0
Serum total protein	17,991	g dl^−1^	6.47	0.38	6.20	6.50	6.70
Serum phospholipid	17,991	mg dl^−1^	280	35	255	278	302
Serum folic acid	17,990	ng ml^−1^	7.24	4.82	3.80	5.60	9.00

	*n*	%					
Marital status	17,702						
Married	17,007	96.1					
Unmarried/single	695	3.9					
Missing	295						
Gestational weight gain (kg)	17,568						
<5	1311	7.5					
5–13	12,216	69.5					
≥13	4041	23.0					
Missing	429						
Education (years in school)	17,634						
≤9	876	5.0					
9–14	12,998	73.7					
≥15	3760	21.3					
Missing	363						
Household income (million yen)	16,488						
<6	12,140	73.6					
≥6	4348	26.4					
Missing	1509						
Smoking during pregnancy	17,810						
No	16,889	94.8					
Yes	921	5.2					
Missing	187						
Passive smoking during pregnancy	17,674						
No	10,862	61.4					
Yes	6812	38.5					
Missing	323						
Alcohol consumption during pregnancy	17,833						
No	17,255	96.8					
Yes	578	3.2					
Missing	164						
Primipara	17,523						
Yes	6927	39.5					
No	10,596	60.5					
Missing	474						
Pregnancy situation	17,997						
Singleton	17,828	99.1					
Multiple	169	0.9					
Study area (Regional centre)	17,997						
Hokkaido	1384	7.7					
Miyagi	1572	8.7					
Fukushima	1968	10.9					
Chiba	1062	5.9					
Kanagawa	1083	6.0					
Koshin (Yamanashi and Shinshu)	1379	7.7					
Toyama	1026	5.7					
Aichi	1074	6.0					
Kyoto	659	3.7					
Osaka	1513	8.4					
Hyogo	967	5.4					
Tottori	586	3.3					
Kochi	1145	6.4					
Fukuoka	1390	7.7					
South Kyushu/Okinawa (Kumamoto, Miyazaki and Okinawa)	1189	6.6

**Table 2:** Summary statistics of Hg, Pb, Cd, Mn and Se concentrations in whole blood samples (*n* = 17,997) collected from JECS mothers during late/mid-term pregnancy (volumetric concentrations^a^)

	Hg µg l^−1^	Pb µg dl^−1^	Cd µg l^−1^	Mn µg l^−1^	Se µg l^−1^
% Detection	100	100	100	100	100
Summary statistics					
Minimum	0.35	0.16	0.10	4.35	105
25th Percentile	2.70	0.51	0.52	13.2	165
Median	3.83	0.63	0.70	16.1	178
75th Percentile	5.43	0.78	0.95	19.6	192
95th Percentile	9.26	1.15	1.55	25.7	217
Maximum	30.6	7.45	4.97	44.5	390
Mean	4.41	0.68	0.79	16.1	180
Standard deviation	2.56	0.30	0.41	4.93	21.5
Geometric mean	3.83	0.64	0.71	16.1	179
95% CI for geometric mean	3.80–3.86	0.63–0.64	0.71–0.72	16.0–16.2	178–179

*Hg* mercury, *Pb* lead, *Cd* cadmium, *Mn* manganese, *Se* selenium, *JECS* Japan Environment and Children’s Study, *95% CI* 95% confidence interval.

^a^1.0506 was used as a typical specific gravity of whole blood to convert gravimetric concentrations to volumetric concentrations.

Figure 1 shows the distribution of the five target elements, all of which exhibited right-skewed distributions. Little correlation among the target elements was observed. The element pairs that showed correlation coefficients >0.2 were Hg–Se (Spearman’s *ρ* *=* 0.287), Pb–Cd (*ρ* *=* 0.239) and Cd–Mn (*ρ* *=* 0.267).

### Potential predictors of the maternal blood concentrations of the target elements

Tables S4–S6 show the associations between the five element concentrations and the maternal characteristics. In general, the associations were weak. The relationships that had absolute values of coefficients of correlation ≥0.1 were: Cd–age at delivery (*ρ* *=* 0.217), Mn–gestational week at sampling (*ρ* *=* 0.273), Se–gestational weight gain (*ρ* *=* 0.115), Hg–tuna consumption (*ρ* *=* 0.214), Hg–seafood consumption (*ρ* *=* 0.187), Hg–dried fish consumption (*ρ* *=* 0.140), Hg–yellowtail tuna consumption (*ρ* *=* 0.174), Hg–horse mackerel and sardine consumption (*ρ* *=* 0.105), Hg–squid consumption (*ρ* *=* 0.112) and Se–tuna consumption (*ρ* *=* 0.104).

Table 3 presents the results of the multiple regression analysis for each element. The adjusted *R*^2^ were 0.149, 0.074, 0.188, 0.087 and 0.137 for Hg, Pb, Cd, Mn and Se, respectively. The largest variance inflation factor (VIF) for each element was 5.2 (maternal education for all element models). Most of the VIFs were below or around 2, which indicated that the collinearity did not affect the models. The highest absolute *β* (|*β|*) for Hg was for the highest quartile of seafood consumption (≥46.0 g day^−1^, *β* *=* 0.301) followed by study area (Kochi, *β* *=* 0.248), maternal education (≥15 years, *β* *=* 0.109) and confectionery consumption (≥31.7 g day^−1^, *β* *=* −0.102). For Pb, the order of |*β|* was as follows: study areas (Toyama, *β* *=* 0.135; South Kyushu/Okinawa, *β* *=* 0.128), maternal age at delivery (*β* *=* 0.117) and consumption of non-alcoholic beverages (≥478 ml day^−1^, *β* *=* 0.105). For Cd, the largest |*β|* was maternal age at delivery (*β* *=* 0.275) followed by smoking during pregnancy (*β* *=* 0.220), study area (Toyama, *β* *=* 0.200) and education (≥15 years, *β* *=* −0.131). The gestational age at sampling had the highest |*β|* (*β* *=* 0.286) for Mn. For Se, serum total protein had the highest |*β|* (*β* *=* 0.161) followed by folic acid consumption (*β* *=* −0.145), study area (Kochi, *β* *=* 0.125) and seafood consumption (≥46.0 g day^−1^, *β* *=* 0.116).

**Table 3:** Multiple regression analysis to identify potential determinants of maternal blood metal concentrations

	Log_10_ (Hg)		Log_10_ (Pb)		Log_10_ (Cd)		Mn		Se	
*N*	15,225		15,225		15,225		15,225		15,219	
Adjusted *R*^2^	0.149		0.074		0.188		0.087		0.137	
*y*-intercept	0.373		0.633		−0.506		5.11		89.0	

Determinants	*β*	SE	*β*	SE	*β*	SE	*β*	SE	*β*	SE
Gestational week at sampling (week)	−0.010	0.009	0.049	0.009	0.064	0.008	0.286	0.009	−0.031	0.009
Maternal age at delivery (years)	−0.002	0.008	0.117	0.009	0.275	0.008	−0.015	0.009	0.064	0.008
Pregnancy status										
Single	Ref		Ref		Ref		Ref		Ref	
Multiple	−0.024	0.008	−0.009	0.008	0.003	0.007	0.028	0.008	−0.022	0.008
Marital status										
Married	Ref		Ref		Ref		Ref		Ref	
Unmarried/single	−0.013	0.008	0.017	0.008	0.013	0.008	−0.011	0.008	0.002	0.008
Education (years in school)										
≤9	Ref		Ref		Ref		Ref		Ref	
9–14	0.073	0.016	−0.031	0.017	−0.108	0.016	−0.012	0.017	0.016	0.017
≥15	0.109	0.017	−0.045	0.018	−0.131	0.017	−0.028	0.018	0.010	0.017
Household income (million yen)										
<6	Ref		Ref		Ref		Ref		Ref	
≥6	0.081	0.008	0.006	0.008	0.000	0.008	−0.020	0.008	−0.005	0.008
Smoking during pregnancy										
No	Ref		Ref		Ref		Ref		Ref	
Yes	−0.007	0.008	0.071	0.008	0.220	0.008	−0.041	0.008	−0.001	0.008
Passive smoking during pregnancy										
No	Ref		Ref		Ref		Ref		Ref	
Yes	0.007	0.008	0.049	0.008	0.030	0.008	−0.007	0.008	−0.005	0.008
Alcohol consumption during pregnancy										
No	Ref		Ref		Ref		Ref		Ref	
Yes	0.011	0.008	0.051	0.008	−0.003	0.007	−0.002	0.008	0.020	0.008
Primipara										
Yes	Ref		Ref		Ref		Ref		Ref	
No	0.003	0.008	−0.068	0.008	−0.053	0.008	0.040	0.008	0.049	0.008
Weight gain during pregnancy (kg)										
<5	Ref		Ref		Ref		Ref		Ref	
5–13	0.000	0.013	−0.016	0.014	0.023	0.013	0.012	0.014	−0.021	0.013
≥13	0.006	0.013	−0.005	0.014	0.046	0.013	0.033	0.014	−0.027	0.014
Study area										
Hokkaido	Ref		Ref		Ref		Ref		Ref	
Miyagi	0.163	0.011	0.089	0.012	0.106	0.011	0.003	0.012	0.093	0.011
Fukushima	0.110	0.012	0.060	0.012	0.059	0.012	0.000	0.012	0.111	0.012
Chiba	0.174	0.010	−0.009	0.010	−0.011	0.010	−0.025	0.010	0.086	0.010
Kanagawa	0.172	0.010	−0.012	0.010	0.022	0.010	−0.008	0.010	0.102	0.010
Koshin	0.186	0.010	0.092	0.011	0.011	0.010	0.009	0.011	0.106	0.011
Toyama	0.108	0.010	0.135	0.010	0.200	0.010	0.000	0.010	0.073	0.010
Aichi	0.135	0.010	0.077	0.011	0.038	0.010	−0.010	0.010	0.119	0.010
Kyoto	0.065	0.009	0.069	0.010	0.048	0.009	0.004	0.009	0.037	0.009
Osaka	0.110	0.011	0.088	0.011	0.029	0.011	−0.011	0.011	0.070	0.011
Hyogo	0.068	0.010	0.071	0.010	0.022	0.010	−0.006	0.010	0.065	0.010
Tottori	0.051	0.009	0.083	0.009	0.022	0.009	−0.017	0.009	0.026	0.009
Kochi	0.248	0.010	0.072	0.010	−0.032	0.010	−0.002	0.010	0.125	0.010
Fukuoka	0.026	0.011	0.061	0.011	−0.020	0.010	−0.014	0.011	0.009	0.011
South Kyushu/Okinawa	0.086	0.010	0.128	0.011	−0.035	0.010	0.007	0.011	0.019	0.010
*Food consumption (quartile or categorical)*										
Grain (g day^−1^)										
<354	Ref		Ref		Ref		Ref		Ref	
354–439	0.015	0.009	−0.020	0.010	0.020	0.009	0.016	0.010	0.022	0.009
439–528	0.032	0.010	−0.029	0.010	0.043	0.009	0.034	0.010	−0.002	0.010
≥528	0.027	0.010	−0.014	0.010	0.072	0.010	0.043	0.010	0.024	0.010
Tubers/starch (g day^−1^)										
<10.7	Ref		Ref		Ref		Ref		Ref	
10.7–19.3	−0.016	0.010	−0.014	0.010	−0.012	0.010	−0.008	0.010	−0.014	0.010
19.3–31.0	−0.043	0.010	−0.021	0.011	−0.003	0.010	0.008	0.011	−0.017	0.010
≥31.0	−0.045	0.011	−0.033	0.012	−0.009	0.011	0.009	0.012	−0.041	0.011
Sugar/sweets (taken during pregnancy)										
No	Ref		Ref		Ref		Ref		Ref	
Yes	−0.018	0.008	0.021	0.008	−0.010	0.008	−0.003	0.008	−0.020	0.008
Beans (g day^−1^)										
<17.2	Ref		Ref		Ref		Ref		Ref	
17.2–34.0	0.026	0.010	−0.007	0.010	−0.003	0.010	−0.007	0.010	0.012	0.010
34.0–64.0	0.017	0.010	0.001	0.011	0.006	0.010	−0.008	0.011	0.006	0.010
≥64.0	0.001	0.011	−0.018	0.011	−0.013	0.011	−0.024	0.011	−0.013	0.011
Nuts (taken during pregnancy)										
No	Ref		Ref		Ref		Ref		Ref	
Yes	−0.006	0.008	0.008	0.009	0.012	0.008	−0.001	0.009	−0.021	0.008
Vegetables (g day^−1^)										
<95.4	Ref		Ref		Ref		Ref		Ref	
95.4–149	−0.018	0.010	−0.005	0.010	0.009	0.010	0.001	0.010	−0.010	0.010
149–223	−0.004	0.011	0.005	0.011	0.007	0.011	−0.014	0.011	−0.010	0.011
≥223	0.008	0.012	0.038	0.013	0.002	0.012	−0.014	0.012	−0.024	0.012
Fruits (g day^−1^)										
<48.8	Ref		Ref		Ref		Ref		Ref	
48.8–109	0.007	0.010	−0.003	0.010	−0.009	0.009	0.003	0.010	−0.006	0.010
109–194	−0.007	0.010	−0.007	0.010	−0.009	0.010	0.019	0.010	−0.011	0.010
≥194	−0.005	0.010	−0.014	0.011	−0.032	0.010	0.013	0.011	−0.009	0.010
Mushrooms (g day^−1^)										
<2.7	Ref		Ref		Ref		Ref		Ref	
2.7–6.4	0.043	0.011	0.002	0.011	0.001	0.010	−0.026	0.011	0.011	0.011
6.4–12.9	0.058	0.011	0.015	0.011	0.013	0.011	−0.034	0.011	0.031	0.011
≥12.9	0.073	0.012	0.016	0.012	0.017	0.012	−0.031	0.012	0.030	0.012
Seaweeds (g day^−1^)										
<1.5	Ref		Ref		Ref		Ref		Ref	
1.5–4.3	0.009	0.010	0.027	0.011	0.010	0.010	−0.006	0.011	−0.015	0.010
4.3–8.3	0.000	0.011	0.038	0.011	0.016	0.010	−0.004	0.011	−0.010	0.011
≥8.3	−0.013	0.011	0.035	0.012	0.031	0.011	0.007	0.012	−0.028	0.011
Seafood (g day^−1^)										
<14.3	Ref		Ref		Ref		Ref		Ref	
14.3–28.0	0.102	0.010	0.008	0.010	0.011	0.009	0.016	0.010	0.025	0.010
28.0–46.0	0.198	0.010	0.005	0.011	0.016	0.010	0.005	0.011	0.069	0.010
≥46.0	0.301	0.011	0.018	0.012	0.014	0.011	−0.006	0.012	0.116	0.011
Meat (g day^−1^)										
<37.7	Ref		Ref		Ref		Ref		Ref	
37.7–59.0	−0.019	0.010	0.003	0.010	−0.022	0.010	0.000	0.010	−0.031	0.010
59.0–89.7	−0.038	0.010	0.020	0.011	−0.025	0.010	0.001	0.011	−0.049	0.011
≥89.7	−0.044	0.012	0.021	0.012	−0.034	0.011	0.011	0.012	−0.071	0.012
Eggs (g day^−1^)										
<10.7	Ref		Ref		Ref		Ref		Ref	
10.7–25.0	−0.007	0.013	−0.006	0.013	−0.008	0.012	−0.001	0.013	−0.015	0.013
25.0–39.3	−0.015	0.013	−0.026	0.014	−0.011	0.013	−0.001	0.014	−0.019	0.013
≥39.3	−0.007	0.013	−0.016	0.014	−0.014	0.013	0.008	0.014	−0.043	0.013
Dairy (g day^−1^)										
<104	Ref		Ref		Ref		Ref		Ref	
104–200	−0.015	0.010	−0.026	0.010	−0.015	0.009	0.001	0.010	−0.029	0.010
200–325	−0.024	0.010	−0.057	0.010	−0.033	0.010	0.010	0.010	−0.036	0.010
≥325	−0.029	0.010	−0.079	0.011	−0.056	0.010	0.002	0.010	−0.071	0.010
Fat (g day^−1^)										
<6.6	Ref		Ref		Ref		Ref		Ref	
6.6–9.7	−0.013	0.010	−0.017	0.011	−0.016	0.010	−0.003	0.011	0.007	0.011
9.7–13.7	−0.027	0.011	−0.018	0.012	−0.009	0.011	0.009	0.012	0.053	0.011
≥13.7	−0.033	0.013	−0.022	0.014	−0.012	0.013	0.011	0.014	0.093	0.013
Confectionery (g day^−1^)										
<9.3	Ref		Ref		Ref		Ref		Ref	
9.3–18.4	−0.045	0.010	−0.018	0.010	−0.017	0.009	−0.014	0.010	−0.012	0.010
18.4–31.7	−0.084	0.010	−0.029	0.010	−0.019	0.010	−0.007	0.010	−0.044	0.010
≥31.7	−0.114	0.010	−0.034	0.011	−0.008	0.010	−0.010	0.011	−0.074	0.010
Non-alcohol beverages (ml day^−1^)										
<126	Ref		Ref		Ref		Ref		Ref	
126–271	0.016	0.010	0.035	0.010	0.020	0.009	−0.002	0.010	0.017	0.010
271–478	0.012	0.010	0.059	0.010	0.012	0.009	−0.003	0.010	0.004	0.010
≥478	0.032	0.010	0.105	0.010	0.021	0.010	0.016	0.010	0.025	0.010
Seasoning/spices (g day^−1^)										
<9.7	Ref		Ref		Ref		Ref		Ref	
9.7–15.0	−0.003	0.010	−0.009	0.010	0.006	0.009	−0.007	0.010	0.017	0.010
15.0–22.0	0.001	0.010	−0.014	0.011	0.005	0.010	−0.027	0.010	0.002	0.010
≥22.0	0.015	0.011	−0.002	0.011	0.029	0.011	−0.032	0.011	−0.011	0.011
Tap/well water (times per week)										
<1	Ref		Ref		Ref		Ref		Ref	
1–2	0.001	0.008	0.012	0.008	−0.008	0.008	−0.007	0.008	−0.015	0.008
≥3	0.006	0.008	0.051	0.008	0.006	0.008	0.009	0.008	−0.011	0.008
Bottled water (times per week)										
<1	Ref		Ref		Ref		Ref		Ref	
1–2	−0.012	0.008	−0.009	0.008	−0.001	0.008	0.008	0.008	0.010	0.008
≥3	−0.004	0.008	−0.020	0.009	−0.028	0.008	−0.002	0.009	0.016	0.008
Serum total protein (g dl^−1^)	–		−		−		−		0.161	0.008
Serum phospholipid (mg dl^−1^)	–		−		−		−		0.144	0.008
Serum folic acid (ng ml^−1^)	–		−		−		−		−0.145	0.008

*Hg* mercury, *Pb* lead, *Cd* cadmium, *Mn* manganese, *Se* selenium, *log*_10_ common logarithm, *R*^2^ coefficient of determination, *β* standardised partial regression coefficient, *SE* standard error, *Ref* reference.

## Discussion

### Correlations among the elements

We found weak correlations in the Hg–Se (Spearman’s *ρ* *=* 0.287), Pb–Cd (*ρ* *=* 0.239) and Cd–Mn (*ρ* *=* 0.267) pairs. A few previous studies reported correlations among these elements in the blood of pregnant women. Ikeda et al. found weak correlations between Pb and Cd (*r* = 0.235) and between Cd and Mn (*r* = 0.168) in Japan [18]. Sun et al. also reported a weak correlation between maternal blood Pb and Cd (*r* = 0.24) in a Chinese study [19]. These previous findings are consistent with our results (small correlation coefficients). The absence or weakness of correlations implies that these elements do not share sources of exposure, i.e., they have different intake routes.

### Mercury

The Japan Food Safety Commission states that the tolerable weekly methylmercury intake is based on the total Hg concentration in maternal hair of 11 ppm, which is derived from the results of the Faroe Islands study and the Seychelles Child Development Study. The Commission then calculated the equivalent total Hg concentration in blood to be 44 µg l^−1^, assuming that the hair:blood Hg ratio is 250:1 [20]. None of the 17,997 maternal samples had Hg concentrations that exceeded 44 µg l^−1^.

The German Human Biomonitoring Commission has also published human biomonitoring (HBM) values [21]. These health-related HBM values are derived from epidemiological studies and are defined as two types: HBM-I and HBM-II. The HBM-I value is the concentration of a substance in a human specimen below which adverse health effects are not expected. The HBM-II value is the concentration above which there is an increased risk of adverse health effects. The HBM-I and -II values for Hg in the blood of children and women of reproductive age are 5 and 15 µg l^−1^, respectively [22]. Of the 17,997 participants, 5372 (29.8%) had Hg concentrations between HBM-I and HBM-II, which is in the range where health effects cannot be excluded with sufficient certainty according to the German HBM Commission. Only 104 participants (0.58%) had Hg concentrations that exceeded HBM-II. The fact that almost 30% of participants fell into the range between HBM-I and II indicates that further studies are required to examine the health effects of relatively low exposure to Hg.

The median and geometric mean Hg level in the 17,997 samples were both 3.83 µg l^−1^ (3.65 ng g^−1^). Compared with other studies, these Hg levels are comparable with the blood Hg levels in pregnant Japanese women in 2006–2007 (median, 3.79 ng g^−1^; *n* = 54) [23] and another group of Japanese pregnant women (geometric mean, 5.18 ng g^−1^; *n* = 115, survey years not specified in the reference) [24]. The blood Hg levels in our study were also similar to those in pregnant Taiwanese women in 2010–2011 (median, 2.24 µg l^−1^; *n* = 145) [25]. However, the blood Hg levels in our study were almost one order of magnitude higher than the blood levels of pregnant women in the USA: Morello-Frosch et al. reported a geometric mean Hg level of 0.45 µg l^−1^ in 2010–2011 (*n* = 77) [26] while Jain reported a geometric mean of 0.706 µg l^−1^ in 2003–2010 (*n* = 735) [27]. Similarly, low levels of blood Hg were also detected in pregnant Canadian women in the third trimester in 2008–2011 (median, 0.56 µg l^−1^; *n* = 1,673) [28] and pregnant Swedish women in 2002–2003 (median, 0.70 µg l^−1^; *n* = 100) [29]. This difference can be attributed to the fact that East Asians tend to eat more fish/seafood than North Americans and Europeans.

The major source of blood Hg in Japan is piscivorous fish [30]. Indeed, the multivariate model showed that of all factors that were examined, seafood consumption contributed the most to the blood Hg levels, with the group with the highest quartile seafood consumption (≥46.0 g day^−1^) having 44% higher blood Hg levels than the lowest quartile group (<14.3 g day^−1^). This is consistent with the findings of other studies [31, 32]. Higher maternal education levels are also associated with increased blood Hg concentrations. It could be explained by the fact that higher the mother’s education levels, the more fish they consumed (data not shown). Some study areas also associated with higher blood Hg levels. However, the study areas did not differ markedly in fish consumption (data not shown). Thus, the association between area and Hg levels may reflect different regional preferences for fish such as tuna. Unlike previous studies [31, 32], we did not observe that maternal age and smoking habits associated with blood Hg levels. This could be due to the fact that the age range of our participants was narrow (mean, 31.2 years; standard deviation, 5.0 years) and very few of the participants smoked during pregnancy (5.2%). It is advised that both epidemiological and benefit-risk modelling are used to develop advice regarding fish consumption for pregnant women [33]. JECS will provide authorities with additional epidemiological evidence to improve fish consumption advice for pregnant women.

### Lead

The HBM values are not set for Pb because the German ‘HBM Commission concluded that establishing an effect threshold for blood Pb levels would be arbitrary and therefore not justified [22]. However, the CDC recommends follow-up blood Pb tests for pregnant women whose blood Pb levels exceed 5 µg dl^−1^ and taking action to reduce exposure to Pb sources [34]. In the current study, five mothers (0.03%) had Pb concentrations above 5 µg dl^−1^.

A study on pregnant Japanese women in 1984 reported that their mean blood Pb level was 8.2 µg dl^−1^ (*n* = 105) [35]. Another study on postpartum Japanese women (on average 2.9 days after birth) in 1988 reported that the geometric mean blood Pb level was 3.6 µg dl^−1^ (*n* = 73) [36]. In our study, the geometric mean Pb level was 0.64 µg dl^−1^. This suggests that the blood Pb levels of pregnant women in Japan have dropped by 5–10-fold in the past 25 years. The National Health and Nutrition Examination Survey (NHANES) has recorded similar declines in blood Pb levels in the general American population [37]. Consequently, the Pb level we detected (median, 0.63 µg dl^−1^) is similar to the Pb levels in women in general from the USA: Morello-Frosch et al. and Jain reported geometric mean Pb levels of 0.65 µg dl^−1^ in 2010–2011 [26] and 0.638 µg dl^−1^ in 2003–2010 [27], respectively. The Pb level in our study was also similar to the Pb levels in pregnant Canadian women studied in 2008–2011 (median, 0.5595 µg dl^−1^) [28] but slightly lower than the median blood Pb levels in pregnant Swedish women in 2002–2003 (1.1 µg dl^−1^) [29].

The Pb model resulted in a low adjusted *R*^2^ (0.074). This suggests there were multiple sources of Pb. To our knowledge, sources of Pb exposure have not been recently identified in Japan. Of the possible sources that were examined, the study area, maternal age and non-alcoholic beverage consumption contributed the most, although their individual effects were small. A previous study has shown that blood Pb concentrations associate with smoking [38]. This association was not observed in this study (*β* = 0.071 and 0.049 for smoking and passive smoking during pregnancy, respectively). In our study, BMI was not included in the model because it related highly with weight gain during pregnancy (data not shown). Wang et al. reported that BMI associated with blood Pb levels [39]. However, in the current study, weight gain during pregnancy had little effect on blood Pb levels (*β* = 0.046 for weight gain >13 kg). Saoudi et al. reported that significant predictors of Pb levels in cord blood were tap water consumption, alcohol consumption, shellfish consumption, vegetable consumption, bread consumption, smoking and the mother being born in countries where lead is often used [15]. However, we did not find any association between blood Pb levels and any of those factors except for non-alcoholic beverages. The correlation between blood Pb levels and non-alcoholic beverage consumption was weak (|*r|* < 0.1). A further study is required to identify the exposure sources of Pb to reduce blood Pb levels in pregnant women.

### Cadmium

The CDC sets a Cd action level of 5 µg l^−1^ for its biomonitoring programme [40]. None of the 17,997 samples in this study exceeded this value, although a few did approach that level: in descending order, the first three highest concentration samples had Cd levels of 4.97, 4.90 and 4.57 µg l^−1^.

One study measured the blood Cd levels in pregnant women in Japan. It was conducted in 1984 and reported a mean Cd concentration of 7 ng g^−1^ [35]. In our study, the mean Cd level was 0.76 ng g^−1^, which is 10 times lower than the level reported previously. However, the Cd level in our participants (geometric mean, 0.71 µg l^−1^; median, 0.70 µg l^−1^) was 2.3–3.5 times higher than the geometric mean Cd levels in pregnant American women that were reported by Morello-Frosch et al. in 2010–2014 (0.22 µg l^−1^) [26] and Jain in 2003–2010 (0.246 µg l^−1^) [27], and the median Cd levels in pregnant Canadian (0.2023 µg l^−1^) [28] and Swedish (0.30 µg l^−1^) women [29]. Notably, the NHANES study of the general American population reports that pregnant non-Hispanic Asian women without a smoking history had higher blood Cd than pregnant women from other ethnic groups [41]. This may reflect higher consumption of rice by Asians: rice is suggested to be a leading source of dietary Cd intake in Japan [42].

The multivariate model suggested that Cd blood increased with age. Smoking also associated with higher Cd levels. These findings agree with those of previous studies [31, 38, 41, 43, 44]. The increase in Cd levels with age is due to the fact that the amount of Cd that the body can eliminate is limited: as a result, Cd has a half-life >26 years for humans [43]. We also found that a longer education is a negative predictor of blood Cd. The reason for this association is unclear but it was also detected by a study on never-pregnant Norwegian women [31]. Rice consumption (which was combined into the grain consumption factor) did not have a large effect on Cd levels in our model (*β* = 0.072) when compared to the effect of smoking (*β* = 0.220). A review article by Ikeda et al. reported that rice consumption accounted for 30–40% of daily Cd intake in non-polluted areas of Japan until 2000 and that the mean Cd content of polished rice is ~50–60 µg/kg, which corresponds to a daily Cd intake of ~30–60 µg/day [45]. According to this report, daily Cd intake in non-polluted areas recently decreased to 11.5 and 16.5 µg/day based on urine and blood Cd concentrations, respectively. Daily intake of Cd from wheat is reported to be one-fifth of that from rice [46]. We combined the consumption of rice with other grains such as wheat, which may have weakened the effect of rice on the blood Cd levels in our model. In addition, given that study areas associated with blood Cd levels in our study, more detailed investigation into the effect of this variable on blood Cd levels is warranted. The blood Cd levels of Japanese pregnant women were low in general. JECS will examine the effects of low levels of Cd on children’s health and development.

### Manganese

Action levels are not available for Mn. The median blood Mn level in our participants was 16.1 µg l^−1^. This is similar to or slightly higher than the median blood Mn levels in pregnant Canadian women in 2008–2011 (12.6 µg l^−1^) [28] and pregnant Australian women in 2008–2011 (6.81 µg l^−1^) [47] but lower than the median Mn levels in pregnant South Korean women in 2007–2011 (21.3 µg l^−1^) [48]. The median Mn levels in our study were also lower than the median Mn levels in pregnant Taiwanese women, as shown by two studies: Huang et al. reported median Mn levels of 44.96 µg l^−1^ in 2010–2011 [25] while Tsai et al. reported median Mn levels of 47.0 µg l^−1^ in 2010–2011 [49]. These differences may stem from the different patterns of food consumption in Japan (e.g., the particular focus on rice, green tea and vegetables) [50].

The adjusted *R*^2^ was also small (0.087) for the Mn model. Blood Mn levels increase as the gestational age rises [51]. In this study, the blood Mn level rose 0.448 µg l^−1^ per gestational week, which is negligible compared with the median level of 16.1 µg l^−1^. No other factors associated significantly with blood Mn levels. This may be because Mn is an essential element and its blood level is very well maintained. The major source of Mn intake is considered to be food; [50] however, no particular food items were a major contributor in our model. Currently, there is no national programme that monitors blood Mn levels in the general population of Japan. Such a programme is warranted because Mn has been reported to affect children’s development [6–9].

### Selenium

The CDC considers 500 µg l^−1^ Se to be an action level [40]. Hays et al. calculated the biomonitoring equivalents (BEs) based on a Chinese cohort study [52]. They presented the BEs of 480 and 400 µg l^−1^ in whole blood based on the Tolerable Upper Intake Level set by the USA National Academy of Medicine (formerly the Institute of Medicine) and the ATSDR Minimal Risk Level, respectively. None of the participants in our study had Se concentrations that exceeded these levels. The median Se level in our participants was 178 µg l^−1^ (169 ng g^−1^). This is similar to the median Se levels in pregnant Chinese women from a study conducted in 2011 (141 ng g^−1^) [53]. Another study on pregnant Chinese women reported similar results (median, 131 ng g^−1^) [19]. Studies conducted in pregnant women in the United Kingdom [54] and Australia [47] reported slightly lower median Se levels of 79.6 and 88.2 µg l^−1^, respectively.

Blood Se concentrations increased by 8.97 µg l^−1^ with every unit (g dl^−1^) increase in serum protein. This approaches half the IQR (13.7 µg l^−1^) in this study but is still small when compared to the difference (283 µg l^−1^) between the 95th percentile (217 µg l^−1^) and the CDC’s action level of 500 µg l^−1^. Study area and seafood consumption also associated with blood Se, like Hg. Food is considered to be a major source of Se intake in Japan [55]. The negative association with folic acid is puzzling because it has not been reported in any previous studies. JECS will further investigate the effect of co-exposure to Se and Hg on children’s neurodevelopment.

### Strength and limitations

The JECS cohort is considered to be representative of the pregnant women in Japan [2]. We randomly selected the 20,000 samples for this study from the whole cohort of JECS. This means we can extrapolate our results to pregnant Japanese women. However, it may not be possible to generalise our findings to the non-pregnant adult female population.

A self-administered questionnaire was used to provide the covariate variable data, including maternal characteristics, food consumption and other exposures such as smoking habits and occupation-related exposures. This approach may have caused considerable uncertainty, especially with regard to the food consumption and smoking habit data, which likely resulted in misclassification. This may have reduced the coefficient of determination in the multivariate models.

## Conclusion

This study presented the distributions of the Hg, Pb, Cd, Mn and Se blood concentrations in Japanese pregnant women. Blood Pb levels have decreased by 5–10-fold over the past 25 years in Japan, as in other developed countries. The main predictors of the blood level of each element were fish consumption for Hg, maternal age and non-alcoholic beverage consumption for Pb, maternal age and smoking for Cd, gestational age at sampling for Mn and serum protein levels for Se.

## Supplementary information

Supplementary Information

Supplementary Figure legends

Supplementary TableS1

Supplementary TableS2

Supplementary TableS3

Supplementary TableS4

Supplementary TableS5

Supplementary TableS6

Supplementary FigureS1

Supplementary FigureS2

Supplementary FigureS3

Supplementary FigureS4

Supplementary FigureS5

## References

[1] Kawamoto T, Nitta H, Murata K, Toda E, Tsukamoto N, Hasegawa M (2014). Rationale and study design of the Japan environment and children’s study (JECS). BMC Public Health.

[2] Michikawa T, Nitta H, Nakayama SF, Yamazaki S, Isobe T, Tamura K (2018). Baseline profile of participants in the Japan Environment and Children’s Study (JECS). J Epidemiol.

[3] Ciesielski T, Weuve J, Bellinger DC, Schwartz J, Lanphear B, Wright RO (2012). Cadmium exposure and neurodevelopmental outcomes in U.S. children. Environ Health Perspect.

[4] Murata K, Iwata T, Dakeishi M, Karita K (2009). Lead toxicity: does the critical level of lead resulting in adverse effects differ between adults and children?. J Occup Health.

[5] Ng DKK, Chan CH, Soo MT, Lee RSY (2007). Low-level chronic mercury exposure in children and adolescents: meta-analysis. Pediatr Int.

[6] Claus Henn B, Ettinger AS, Schwartz J, Téllez-Rojo MM, Lamadrid-Figueroa H, Hernández-Avila M (2010). Early postnatal blood manganese levels and children’s neurodevelopment. Epidemiology.

[7] Claus Henn B, Schnaas L, Ettinger AS, Schwartz J, Lamadrid-Figueroa H, Hernández-Avila M (2012). Associations of early childhood manganese and lead coexposure with neurodevelopment. Environ Health Perspect.

[8] Gunier RB, Arora M, Jerrett M, Bradman A, Harley KG, Mora AM (2015). Manganese in teeth and neurodevelopment in young Mexican-American children. Environ Res.

[9] Lin CC, Chen YC, Su FC, Lin CM, Liao HF, Hwang YH (2013). In utero exposure to environmental lead and manganese and neurodevelopment at 2 years of age. Environ Res.

[10] Taylor D, Dalton C, Hall A, Woodroofe MN, Gardiner PHE (2009). Recent developments in selenium research. Br J Biomed Sci.

[11] Zhang H, Feng X, Chan HM, Larssen T (2014). New insights into traditional health risk assessments of mercury exposure: Implications of selenium. Environ Sci Technol.

[12] Currie LA (1999). Detection and quantification limits: origins and historical overview. Anal Chim Acta.

[13] Trudnowski RJ, Rico RC (1974). Specific gravity of blood and plasma at 4 and 37 °C. Clin Chem.

[14] Yokoyama Y, Takachi R, Ishihara J, Ishii Y, Sasazuki S, Sawada N (2016). Validity of short and long self-administered food frequency questionnaires in ranking dietary intake in middle-aged and elderly japanese in the Japan Public Health Center-Based Prospective Study for the Next Generation (JPHC-NEXT) Protocol Area. J Epidemiol.

[15] Saoudi A, Dereumeaux C, Goria S, Berat B, Brunel S, Pecheux M (2018). Prenatal exposure to lead in France: Cord-blood levels and associated factors: results from the perinatal component of the French Longitudinal Study since Childhood (Elfe). Int J Hyg Environ Health.

[16] Wasserstein RL, Lazar NA (2016). The ASA’s statement on *p* -values: context, process, and purpose. Am Stat.

[17] R Core Team. R: A Language and Environment for Statistical Computing 2018.

[18] Ikeda M, Ohashi F, Fukui Y, Sakuragi S, Moriguchi J (2011). Cadmium, chromium, lead, manganese and nickel concentrations in blood of women in non-polluted areas in Japan, as determined by inductively coupled plasma-sector field-mass spectrometry. Int Arch Occup Environ Health.

[19] Sun H, Chen W, Wang D, Jin Y, Chen X, Xu Y (2014). The effects of prenatal exposure to low-level cadmium, lead and selenium on birth outcomes. Chemosphere.

[20] Japan Food Safety Commission. Food safety risk assessment related to methylmercury in seafood 2005. https://www.fsc.go.jp/english/topics/methylmercury_risk_assessment.pdf. Accessed 10 January 2019.

[21] Schulz C, Angerer J, Ewers U, Kolossa-Gehring M (2007). The german human biomonitoring commission. Int J Hyg Environ Health.

[22] Schulz C, Wilhelm M, Heudorf U, Kolossa-Gehring M (2012). Reprint of ‘Update of the reference and HBM values derived by the German Human Biomonitoring Commission’. Int J Hyg Environ Health.

[23] Sakamoto M, Murata K, Domingo JL, Yamamoto M, Oliveira RB, Kawakami S (2016). Implications of mercury concentrations in umbilical cord tissue in relation to maternal hair segments as biomarkers for prenatal exposure to methylmercury. Environ Res.

[24] Sakamoto M, Kaneoka T, Murata K, Nakai K, Satoh H, Akagi H (2007). Correlations between mercury concentrations in umbilical cord tissue and other biomarkers of fetal exposure to methylmercury in the Japanese population. Environ Res.

[25] Huang SH, Weng KP, Lin CC, Wang CC, Lee CTC, Ger LP (2017). Maternal and umbilical cord blood levels of mercury, manganese, iron, and copper in southern Taiwan: a cross-sectional study. J Chinese Med Assoc.

[26] Morello-Frosch R, Cushing LJ, Jesdale BM, Schwartz JM, Guo W, Guo T (2016). Environmental chemicals in an urban population of pregnant women and their newborns from San Francisco. Environ Sci Technol.

[27] Jain RB (2013). Effect of pregnancy on the levels of blood cadmium, lead, and mercury for females aged 17-39 years old: data from National Health And Nutrition Examination Survey 2003-2010. J Toxicol Environ Health A.

[28] Arbuckle TE, Liang CL, Morisset AS, Fisher M, Weiler H, Cirtiu CM (2016). Maternal and fetal exposure to cadmium, lead, manganese and mercury: The MIREC study. Chemosphere.

[29] Gerhardsson L, Lundh T (2010). Metal concentrations in blood and hair in pregnant females in Southern Sweden. J Environ Health.

[30] Yaginuma-Sakurai K, Shimada M, Ohba T, Nakai K, Suzuki K, Kurokawa N (2009). Assessment of exposure to methylmercury in pregnant Japanese women by FFQ. Public Health Nutr.

[31] Fløtre CH, Varsi K, Helm T, Bolann B, Bjørke-Monsen AL (2017). Predictors of mercury, lead, cadmium and antimony status in Norwegian never-pregnant women of fertile age. PLoS One.

[32] Birgisdottir BE, Knutsen HK, Haugen M, Gjelstad IM, Jenssen MTS, Ellingsen DG (2013). Essential and toxic element concentrations in blood and urine and their associations with diet: Results from a Norwegian population study including high-consumers of seafood and game. Sci Total Environ.

[33] Groth E (2017). Scientific foundations of fish-consumption advice for pregnant women: epidemiological evidence, benefit-risk modeling, and an integrated approach. Environ Res.

[34] Centers for Disease Control and Prevention. Guidelines for the identification and management of lead exposure in pregnant and lactating women recommendations from the advisory committee on childhood lead poisoning prevention 2010. https://www.cdc.gov/nceh/lead/publications/leadandpregnancy2010.pdf. Accessed 10 January 2019.

[35] Tsuchiya H, Mitani K, Kodama K, Nakata T (1984). Placental transfer of heavy metals in Normal pregnent Japanese women_1984.pdf. Arch Environ Health.

[36] Ohara A, Michitsuji H, Yamana M, Yamaguchi K, Fujiki Y (1988). Blood lead levels in the mothers and their neonates. Sangyo Igaku.

[37] Centers for Disease Control and Prevention. Fourth National Report on human exposure to environmental chemicals, updated tables, September 2013. 2013. https://www.cdc.gov/exposurereport/pdf/FourthReport_UpdatedTables_Volume1_Jan2017.pdf. Accessed 10 January 2019.

[38] Richter PA, Bishop EE, Wang J, Swahn MH (2009). Tobacco smoke exposure and levels of urinary metals in the U.S. youth and adult population: The National Health and Nutrition Examination Survey (NHANES) 1999-2004. Int J Environ Res Public Health.

[39] Wang N, Chen C, Nie X, Han B, Li Q, Chen Y (2015). Blood lead level and its association with body mass index and obesity in China - Results from SPECT-China study. Sci Rep.

[40] Centers for Disease Control and Prevention. Laboratory procedure manual: cadmium, lead, manganese, mercury, and selenium 2014. https://www.cdc.gov/Nchs/Data/Nhanes/Nhanes_13_14/PbCd_H_MET.pdf. Accessed 10 January 2019.

[41] Aoki Y, Yee J, Mortensen ME (2017). Blood cadmium by race/hispanic origin: the role of smoking. Environ Res.

[42] Ikeda M, Shimbo S, Watanabe T, Ohashi F, Fukui Y, Sakuragi S (2011). Estimation of dietary Pb and Cd intake from Pb and Cd in blood or urine. Biol Trace Elem Res.

[43] Agency for Toxic Substances and Disease Registry. Toxicological Profile for Cadmium (update) 2012. https://www.atsdr.cdc.gov/toxprofiles/tp5.pdf. Accessed 10 January 2019. 24049863

[44] Mortensen ME, Wong LY, Osterloh JD (2011). Smoking status and urine cadmium above levels associated with subclinical renal effects in U.S. adults without chronic kidney disease. Int J Hyg Environ Health.

[45] Ikeda M, Watanabe T, Nakatsuka H, Moriguchi J, Sakuragi S, Ohashi F (2015). Cadmium exposure in general populations in Japan: a review. Food Saf.

[46] Shimbo S, Zhang ZW, Watanabe T, Nakatsuka H, Matsuda-Inoguchi N, Higashikawa K (2001). Cadmium and lead contents in rice and other cereal products in Japan in 1998-2000. Sci Total Environ.

[47] Callan AC, Hinwood AL, Ramalingam M, Boyce M, Heyworth J, McCafferty P (2013). Maternal exposure to metals-concentrations and predictors of exposure. Environ Res.

[48] Chung SE, Cheong HK, Ha EH, Kim BN, Ha M, Kim Y (2015). Maternal blood manganese and early neurodevelopment: The mothers and children’s environmental health (MOCEH) study. Environ Health Perspect.

[49] Tsai MS, Liao KW, Chang CH, Chien LC, Mao IF, Tsai YA (2015). The critical fetal stage for maternal manganese exposure. Environ Res.

[50] Yamada M, Asakura K, Sasaki S, Hirota N, Notsu A, Todoriki H (2014). Estimation of intakes of copper, zinc, and manganese in Japanese adults using 16-day semi-weighed diet records. Asia Pac J Clin Nutr.

[51] Takser L, Lafond J, Bouchard M, St-Amour G, Mergler D (2004). Manganese levels during pregnancy and at birth: relation to environmental factors and smoking in a Southwest Quebec population. Environ Res.

[52] Hays SM, Macey K, Nong A, Aylward LL (2014). Biomonitoring Equivalents for selenium. Regul Toxicol Pharmacol.

[53] Hu X, Zheng T, Cheng Y, Holford T, Lin S, Leaderer B (2016). Distributions of heavy metals in maternal and cord blood and the association with infant birth weight in China. J Reprod Med.

[54] Mistry HD, Gill CA, Kurlak LO, Seed PT, Hesketh JE, Méplan C (2015). Association between maternal micronutrient status, oxidative stress, and common genetic variants in antioxidant enzymes at 15 weeks’gestation in nulliparous women who subsequently develop preeclampsia. Free Radic Biol Med.

[55] Ministry of Health, Labour and Welfare. Dietary reference intakes for Japanese (2015). 2018. http://www.mhlw.go.jp/shingi/2009/05/s0529-4.html.

