# Supplementary material for: Blood mercury, lead, cadmium, manganese and selenium levels in pregnant women and their determinants: the Japan Environment and Children’s Study (JECS)
Source: J Expo Sci Environ Epidemiol. 2019 Apr 18;29(5):633–47. doi: 10.1038/s41370-019-0139-0 (PMC6760604; doi:10.1038/s41370-019-0139-0)
Supplement: Supplementary file 1 — Supplementary Information [file 41370_2019_139_MOESM1_ESM.docx]

**Supplementary Information**

**Blood mercury, lead, cadmium, manganese and selenium levels in pregnant women and their determinants: the Japan Environment and Children’s Study (JECS)**

**Authors**

Shoji F. Nakayama^1^, Miyuki Iwai-Shimada^1^, Tomoko Oguri^1, 2^, Tomohiko Isobe^1^, Ayano Takeuchi^1, 3^, Yayoi Kobayashi^1^, Takehiro Michikawa^1, 4^, Shin Yamazaki^1^, Hiroshi Nitta^1^, Toshihiro Kawamoto^1^, and the Japan Environment and Children’s Study Group^5^

**Affiliations**

^1^ Japan Environment and Children’s Study Programme Office, National Institute for Environmental Studies, 16-2 Onogawa, Tsukuba, Ibaraki 305-8506, Japan

^2^ Research Institute of Science for Safety and Sustainability, National Institute of Advanced Industrial Science and Technology, 16-1 Onogawa, Tsukuba, Ibaraki 305-8569, Japan

^3^ Department of Preventive Medicine and Public Health, Keio University, 35 Shinanomachi, Shinjukuku, Tokyo 106-8582, Japan

^4^ Department of Environmental and Occupational Health, School of Medicine, Toho University, 5-21-16 Omori-nishi, Otaku, Tokyo 143-8540, Japan

^5^ The Study Group members are listed in the Appendix

**Corresponding author:**

Shoji F. Nakayama, MD, PhD

Japan Environment and Children’s Study Programme Office, National Institute for Environmental Studies

16-2 Onogawa, Tsukuba, Ibaraki 305-8506, Japan

Telephone: +81 (29) 850-2786

E-mail: fabre@nies.go.jp

1. **Materials and methods**
   1. *Solution preparation*

A standard stock solution containing all the target elements (except Hg) was prepared in 0.14 M nitric acid. A standard stock solution of Hg was generated by mixing the Hg standard solution with 0.056 M nitric acid, 0.5% (w/v) EDTA and 1% (v/v) TMAH. The stock solutions were mixed together to obtain a stock calibration solution in which the Hg, Pb, Cd, Mn and Se concentrations were 200, 200, 20, 600 and 2,000 ng g^−1^, respectively. An internal standard mixture containing Y, In and Tl (each at 250 ng g^−1^) was prepared in the same manner. A dilution solution containing 2% v/v butan-1-ol, 0.1% TMAH, 0.5 g l^−1^ POE and 0.5 g l^−1^ EDTA was prepared on each day of the analysis. A series of calibration solutions was made using the stock calibration solution, the internal standard mixture and the dilution solution to obtain the calibration curves shown in Table S1.

- 1. *Calculation of element concentrations*

Hg concentrations were calculated as total Hg. The intensity of Pb isotopes was the sum of the intensities of ^206^Pb, ^207^Pb and ^208^Pb. To correct for spectral overlap from molybdenum oxide (^95^Mo^16^O), ^111^Cd intensity was calculated using the following equation:

[Cd] = [m/z 111 intensity] − [m/z 95 intensity] × [^95^Mo^16^O generation rate].

The ^95^Mo^16^O generation rate was derived from the following equation:

[^95^Mo^16^O generation rate] = [the intensity of m/z 111 when 100 ppb ^95^Mo standard solution was analysed] / [the intensity of m/z 95 when 100 ppb ^95^Mo standard solution was analysed].

1. **Results**
   1. *Performance characteristics*

The performance characteristics of the ICP-MS method that we developed are shown in Table S1.

- 1. *Method robustness*
     1. *Method comparison*

Total Hg was analysed by cold vapour atomic absorption spectroscopy (CVAAS) as a ‘gold-standard’ method. Pb, Cd, Mn and Se were measured by the acid digestion method using ICP-atomic emission spectroscopy (AES) as a ‘gold-standard’ method. Figure S1 shows Bland-Altman plots comparing the ‘gold-standard’ methods and the current method. The current method was comparable with the traditional methods.

- - 1. *Mercury*

Analysis of Hg was hampered by two problems: 1) instability of Hg in solution and 2) carryover of Hg on instruments. Use of a high pH and EDTA helped to prevent Hg from vaporising. The second problem was solved using two rinse solutions. The first rinse solution was water containing 1 mg ml^−1^ L-cysteine and was used to wash the ICP-MS auto-sampler. The second rinse solution was water containing 2% butan-1-ol, 1% TMAH, 0.5 g l^−1^ POE and 0.5 g l^−1^ H_4_EDTA and was used to washout a sample prior to analysis of the next sample. This method reduced Hg carryover to negligible levels.

- - 1. *Selenium*

ICP-MS is susceptible to polyatomic interference. Polyatomic ions such as ^40^Ar^37^Cl^+^, ^38^Ar^40^Ar^+^ and ^40^Ar^40^Ar^+^ can interfere with signals of the isotopes ^77^Se, ^78^Se and ^80^Se. This can be circumvented by introducing a diatomic gas (e.g., N^2^) or an extra carbon source (e.g., organic solvents) into the plasma [1, 2]. Delvis and Sieniawska reported that addition of butan-1-ol into a sample solution prevents polyatomic interference and blockage of the ICP torch injector tube for more than 6 hours of operation [3]. This procedure was incorporated into our method. Additionally, the Se signal gradually decreased over the course of less than 5 hours of analysis (Figure S2: Room temperature). This problem was solved by ensuring samples were not left on the auto-sampler tray at room temperature for more than 2 hours and by keeping the other samples refrigerated (Figure S2: Refrigerated).

- - 1. *Manganese*

^39^K^16^O and ^54^Fe^1^H can interfere with measurement of ^55^Mn. Whole blood samples contain a significant amount of potassium, which may form ^39^K^16^O and overlap with the reading of ^55^Mn. We analysed a solution containing 100 µg g^−1^ potassium by ICP-MS and monitored the intensity of m/z 55. This intensity was 8,000-fold lower in the potassium solution than in whole blood samples, which was considered negligible. Iron is also abundant in blood samples. We measured Mn in Seronorm^TM^ Trace Elements Whole Blood L-1 by electrothermal atomic absorption spectrometry (ETAAS), which was considered to experience little or no interference by ^54^Fe^1^H, at four independent laboratories. The mean Mn concentration was 19.5 ng g^−1^ (95% confidence interval (CI): 17.7–21.4 ng g^−1^). The same sample was analysed by the current method, which yielded a concentration of 18.8 ng g^−1^ (95% CI: 17.9–19.7 ng g^−1^). The two measurements did not significantly differ; therefore, we concluded that there was little or no interference by ^54^Fe^1^H on Mn measurements using the current method.

- - 1. *Stability*

Stability of the calibration solutions was tested by analysing a series of calibration solutions on day 0, 5, 8 and 14. The calibration solutions were stable for at least 14 days (data not shown). We decided to make the calibration solutions fresh every week. Thaw-and-freeze stability was examined by thawing and freezing the same samples multiple times. The results did not change in four trials over 7 days (data not shown). For QC of day-to-day analysis, we used the Shewhart control chart ($\bar{X}$-*R*_m_ chart) in accordance with ISO 7870. Sample $\bar{X}$-*R*_m_ charts are shown in Figures S3 and S4.

- - 1. *External quality control*

QC sample measurements in three contract laboratories were in good agreement, with differences of less than 10% RSD between each measurement (Figure S5). All three laboratories also joined the external quality control programme (i.e., German External Quality Assessment Scheme (G-EQUAS)) and proved that their results were within the tolerance ranges for all measured elements.

**References**

1. Evans EH, Ebdon L. Simple Approach to Reducing Polyatomic Ion Interferences on Arsenic and Selenium in Inductively Coupled Plasma Mass-Spectrometry. Journal of Analytical Atomic Spectrometry. 1989;4(3):299-300.
2. Larsen EH, Sturup S. Carbon-Enhanced Inductively-Coupled Plasma-Mass Spectrometric Detection of Arsenic and Selenium and Its Application to Arsenic Speciation. Journal of Analytical Atomic Spectrometry. 1994;9(10):1099-105.
3. Delves HT, Sieniawska CE. Simple method for the accurate determination of selenium in serum by using inductively coupled plasma mass spectrometry. Journal of Analytical Atomic Spectrometry. 1997;12(3):387-9.

Figure Caption

Figure S1: Bland-Altman plots comparing gold-standard methods and the current method.

(A) CVAAS (gold-standard) vs. ICP-MS (current) for total mercury. Acid digestion (gold-standard) vs. alkaline dilution (current) for (B) lead, (C) cadmium, (D) manganese and (E) selenium.

Figure S2: Se stability over time after sample preparation.

The intensity of Se at time 0 was used as a reference for each condition.

*Abbreviations*: Se, selenium; Y, yttrium; Se/Y, the intensity of selenium divided by that of yttrium.

Figure S3: $\bar{X}$ charts for (A) mercury, (B) lead, (C) cadmium, (D) manganese and (E) selenium.

Figure S4: *R*_m_ charts for (A) mercury, (B) lead, (C) cadmium, (D) manganese and (E) selenium.

Figure S5: Boxplots of quality control sample measurements in three contract laboratories (A, B and C).

(A) Mercury, (B) lead, (C) cadmium, (D) manganese and (E) selenium.
