# Supplementary material for: Blood mercury, lead, cadmium, manganese and selenium levels in pregnant women and their determinants: the Japan Environment and Children’s Study (JECS)
Source: J Expo Sci Environ Epidemiol. 2019 Apr 18;29(5):633–47. doi: 10.1038/s41370-019-0139-0 (PMC6760604; doi:10.1038/s41370-019-0139-0)
Supplement: Supplementary file 2 — Supplementary Figure legends [file 41370_2019_139_MOESM2_ESM.docx]

**Supplementary Figure Legends**

Figure S1: Bland-Altman plots comparing gold-standard methods and the current method.

(A) CVAAS (gold-standard) vs. ICP-MS (current) for total mercury. Acid digestion (gold-standard) vs. alkaline dilution (current) for (B) lead, (C) cadmium, (D) manganese and (E) selenium.

Figure S2: Se stability over time after sample preparation.

The intensity of Se at time 0 was used as a reference for each condition.

*Abbreviations*: Se, selenium; Y, yttrium; Se/Y, the intensity of selenium divided by that of yttrium.

Figure S3: $\bar{X}$ charts for (A) mercury, (B) lead, (C) cadmium, (D) manganese and (E) selenium.

Figure S4: *R*_m_ charts for (A) mercury, (B) lead, (C) cadmium, (D) manganese and (E) selenium.

Figure S5: Boxplots of quality control sample measurements in three contract laboratories (A, B and C).

(A) Mercury, (B) lead, (C) cadmium, (D) manganese and (E) selenium.
