# Supplementary material for: Blood mercury, lead, cadmium, manganese and selenium levels in pregnant women and their determinants: the Japan Environment and Children’s Study (JECS)
Source: J Expo Sci Environ Epidemiol. 2019 Apr 18;29(5):633–47. doi: 10.1038/s41370-019-0139-0 (PMC6760604; doi:10.1038/s41370-019-0139-0)
Supplement: Supplementary file 3 — Supplementary TableS1 [file 41370_2019_139_MOESM3_ESM.docx]

Table S1. Performance characteristics of the developed method

|  | Repeatability | | | | Intermediate precision | | Calibration (seven points) | | MDL |
| --- | --- | --- | --- | --- | --- | --- | --- | --- | --- |
|  | Reference standard (*n* = 7) | | Pooled QC blood (*n* = 60) | | Seronorm L-1 (*n* = 40) | | Range | *r^2^* |  |
|  | mean, ng g^−1^ | %RSD | mean, ng g^−1^ | %RSD | mean, ng g^−1^ | %RSD | ng g^−1^ |  | ng g^−1^ |
| ^202^Hg | 0.461 | 1.8 | 9.20 | 1.6 | 1.50 | 2.5 | 0–40 | 0.99999 | 0.049 |
| ^206+207+208^ Pb | 0.500 | 2.2 | 12.9 | 0.82 | 10.0 | 1.2 | 0–40 | 0.99999 | 0.129 |
| ^111^Cd | 0.0493 | 1.1 | 0.783 | 1.7 | 0.314 | 3.5 | 0–4 | 0.99995 | 0.023 |
| ^55^Mn | 0.478 | 0.63 | 8.49 | 3.4 | 19.1 | 1.4 | 0–160 | 0.99996 | 0.522 |
| ^78^Se | 4.87 | 1.4 | 194 | 1.4 | 54.9 | 0.89 | 0–400 | 0.99999 | 0.837 |

*Abbreviations*: MDL, method detection limit; QC, quality control; SD, standard deviation; RSD, relative standard deviation; Hg, mercury; Pb, lead; Cd, cadmium; Mn, manganese; Se, selenium.
