# Supplementary material for: Blood mercury, lead, cadmium, manganese and selenium levels in pregnant women and their determinants: the Japan Environment and Children’s Study (JECS)
Source: J Expo Sci Environ Epidemiol. 2019 Apr 18;29(5):633–47. doi: 10.1038/s41370-019-0139-0 (PMC6760604; doi:10.1038/s41370-019-0139-0)
Supplement: Supplementary file 4 — Supplementary TableS2 [file 41370_2019_139_MOESM4_ESM.docx]

Table S2. Operating conditions of the ICP-MS instruments

| Instrument | Agilent 7700 series | |
| --- | --- | --- |
| ICP | RF power | 1.55 kW |
|  | Plasma gas | Argon |
|  | Plasma gas flow rate | 15.0 l min^−1^ |
|  | Carrier gas flow rate | 1.09 l min^−1^ |
|  | S/C Temperature | 2°C |
|  | Reaction gas | H_2_, He |
|  | Reaction gas flow rate | 6 ml min^−1^ for H_2_ |
|  |  | 4.3 ml min^−1^ for He |
| MS | Scan mass range | ^55^Mn, ^77^Se, ^78^Se, ^89^Y, ^95^Mo, ^111^Cd, ^114^Cd, ^115^In, ^206^Pb, ^207^Pb, ^208^Pb, ^200^Hg, ^201^Hg, ^202^Hg, ^205^Tl |
|  | Ion count acquisition (points) | 3 |
|  | Number of replicates | 3 |

*Abbreviations*: ICP-MS, inductively coupled plasma-mass spectrometry.
