# Supplementary material for: Blood mercury, lead, cadmium, manganese and selenium levels in pregnant women and their determinants: the Japan Environment and Children’s Study (JECS)
Source: J Expo Sci Environ Epidemiol. 2019 Apr 18;29(5):633–47. doi: 10.1038/s41370-019-0139-0 (PMC6760604; doi:10.1038/s41370-019-0139-0)
Supplement: Supplementary file 5 — Supplementary TableS3 [file 41370_2019_139_MOESM5_ESM.docx]

Table S3. Summary of Hg, Pb, Cd, Mn and Se concentrations in whole blood samples (*n* = 17,997) collected from JECS mothers during late/mid-term pregnancy (gravimetric concentrations).

|  | Hg | Pb | Cd | Mn | Se |
| --- | --- | --- | --- | --- | --- |
|  | ng g^−1^ | ng g^−1^ | ng g^−1^ | ng g^−1^ | ng g^−1^ |
| % Detection | 100 | 100 | 100 | 100 | 100 |
| Summary statistics |  |  |  |  |  |
| Minimum | 0.33 | 1.50 | 0.10 | 4.14 | 99.9 |
| 25th Percentile | 2.57 | 4.81 | 0.50 | 12.6 | 157 |
| Median | 3.65 | 5.96 | 0.66 | 15.3 | 169 |
| 75th Percentile | 5.17 | 7.46 | 0.91 | 18.7 | 183 |
| 95th Percentile | 8.82 | 10.9 | 1.48 | 24.5 | 207 |
| Maximum | 30.1 | 70.9 | 4.73 | 42.4 | 371 |
| Mean | 4.20 | 6.47 | 0.76 | 16.0 | 171 |
| Standard deviation | 2.44 | 2.84 | 0.39 | 4.70 | 20.5 |
| Geometric mean | 3.65 | 6.05 | 0.68 | 15.3 | 170 |
| 95% CI for geometric mean | 3.62–3.67 | 6.02–6.08 | 0.67–0.68 | 15.2–15.4 | 170–170 |

*Abbreviations:* Hg, mercury; Pb, lead; Cd, cadmium; Mn, manganese; Se, selenium; JECS, Japan Environment and Children’s Study; 95% CI, 95% confidence interval.
