# Supplementary material for: Blood mercury, lead, cadmium, manganese and selenium levels in pregnant women and their determinants: the Japan Environment and Children’s Study (JECS)
Source: J Expo Sci Environ Epidemiol. 2019 Apr 18;29(5):633–47. doi: 10.1038/s41370-019-0139-0 (PMC6760604; doi:10.1038/s41370-019-0139-0)
Supplement: Supplementary file 6 — Supplementary TableS4 [file 41370_2019_139_MOESM6_ESM.docx]

Table S4. Associations between blood metal concentrations and maternal characteristics (continuous variables)

|  |  | Hg | Pb | Cd | Mn | Se |
| --- | --- | --- | --- | --- | --- | --- |
| Gestational day at sampling (weeks) | N | 17884 | 17884 | 17884 | 17884 | 17884 |
|  | *rho* | -0.003 | -0.003 | 0.075 | 0.277 | 0.021 |
|  | *P* | 0.693 | 0.682 | <0.0001 | <0.0001 | 0.005 |
| Age at delivery (years) | N | 17932 | 17932 | 17932 | 17932 | 17932 |
|  | *rho* | 0.059 | 0.067 | 0.229 | -0.028 | 0.051 |
|  | *P* | <0.0001 | <0.0001 | <0.0001 | <0.0001 | <0.0001 |
| Body weight before pregnancy (kg) | N | 17983 | 17983 | 17983 | 17983 | 17983 |
|  | *rho* | 0.025 | 0.073 | 0.045 | 0.054 | 0.064 |
|  | *P* | <0.0001 | <0.0001 | <0.0001 | <0.0001 | <0.0001 |
| Serum total protein (g dl^-1^) | N | 17991 | 17991 | 17991 | 17991 | 17991 |
|  | *rho* | 0.015 | 0.044 | 0.003 | 0.092 | 0.193 |
|  | *P* | 0.042 | <0.0001 | 0.735 | <0.0001 | <0.0001 |
| Serum albumin (g dl^-1^) | N | 17991 | 17991 | 17991 | 17991 | 17991 |
|  | *rho* | 0.015 | 0.012 | -0.065 | -0.072 | 0.164 |
|  | *P* | 0.049 | 0.095 | <0.0001 | <0.0001 | <0.0001 |
| Serum phospholipid (mg dl^-1^) | N | 17991 | 17991 | 17991 | 17991 | 17991 |
|  | *rho* | 0.020 | 0.010 | 0.037 | 0.093 | 0.184 |
|  | *P* | 0.007 | 0.192 | <0.0001 | <0.0001 | <0.0001 |
| Serum folic acid (ng ml^-1^) | N | 17991 | 17991 | 17991 | 17991 | 17991 |
|  | *rho* | 0.017 | -0.065 | -0.031 | -0.033 | -0.188 |
|  | *P* | 0.023 | <0.0001 | <0.0001 | <0.0001 | <0.0001 |
| Mn intake (mg day^-1^) | N | 17719 | 17719 | 17719 | 17719 | 17719 |
|  | *rho* | 0.090 | 0.049 | 0.053 | 0.020 | 0.005 |
|  | *P* | <0.0001 | <0.0001 | <0.0001 | 0.007 | 0.513 |
| Se intake (microg/day^-1^) | N | 17719 | 17719 | 17719 | 17719 | 17719 |
|  | *rho* | 0.091 | -0.013 | 0.012 | -0.010 | 0.089 |
|  | *P* | <0.0001 | 0.095 | 0.113 | 0.201 | <0.0001 |

*Abbreviations:* Hg, mercury; Pb, lead; Cd, cadmium; Mn, manganese; Se, selenium.

Spearman's correlation coefficients (*rho)* were calculated.
