# Supplementary material for: Blood mercury, lead, cadmium, manganese and selenium levels in pregnant women and their determinants: the Japan Environment and Children’s Study (JECS)
Source: J Expo Sci Environ Epidemiol. 2019 Apr 18;29(5):633–47. doi: 10.1038/s41370-019-0139-0 (PMC6760604; doi:10.1038/s41370-019-0139-0)
Supplement: Supplementary file 7 — Supplementary TableS5 [file 41370_2019_139_MOESM7_ESM.docx]

Table S5. Associations between blood metal concentrations and maternal characteristics (categorical variables)

|  | |  | Hg |  | Pb |  | Cd |  | Mn |  | Se |  |
| --- | --- | --- | --- | --- | --- | --- | --- | --- | --- | --- | --- | --- |
|  | | N | *Mean* | *P* | *Mean* | *P* | *Mean* | *P* | *Mean* | *P* | *Mean* | *P* |
| Marital status Married | | 17007 | 4.21 | Ref | 6.46 | Ref | 0.75 | Ref | 16.0 | Ref | 171 | Ref |
| Unmarried/single | | 695 | 3.80 | <0.0001 | 6.72 | 0.0001 | 0.77 | 0.5128 | 15.8 | 0.4015 | 169 | 0.0149 |
| Gestational weight gain (kg) <5 | | 1311 | 4.41 | Ref | 6.64 | Ref | 0.76 | Ref | 15.8 | Ref | 173 | Ref |
| <13 | | 12216 | 4.21 | 0.9994 | 6.46 | 0.0495 | 0.75 | 0.9514 | 15.9 | 0.5079 | 171 | 0.0260 |
| 13≤ | | 4041 | 4.11 | 0.0455 | 6.50 | 0.7949 | 0.77 | 0.0923 | 16.2 | 0.0045 | 171 | 0.0279 |
| Education (years in school) ≤9 | | 876 | 3.85 | Ref | 6.80 | Ref | 0.89 | Ref | 16.1 | Ref | 171 | Ref |
| ≤14 | | 12998 | 4.15 | <0.0001 | 6.48 | <0.0001 | 0.75 | <0.0001 | 16.0 | 0.6715 | 171 | 0.7927 |
| 15≤ | | 3760 | 4.47 | <0.0001 | 6.38 | <0.0001 | 0.73 | <0.0001 | 15.7 | 0.4214 | 170 | 0.2224 |
| Household income (million yens) <6 | | 12140 | 4.07 | Ref | 6.47 | Ref | 0.75 | Ref | 16.0 | Ref | 172 | Ref |
| ≥6 | | 4348 | 4.58 | <0.0001 | 6.48 | 0.5050 | 0.77 | <0.0001 | 15.8 | 0.0048 | 171 | 0.0941 |
| Smoking during pregnancy No | | 16889 | 4.21 | Ref | 6.41 | Ref | 0.73 | Ref | 16.0 | Ref | 171 | Ref |
| Yes | | 921 | 4.03 | <0.0001 | 7.57 | <0.0001 | 1.19 | <0.0001 | 15.3 | <0.0001 | 172 | 0.4530 |
| Passive smoking during pregnancy No | | 10862 | 4.22 | Ref | 6.34 | Ref | 0.73 | Ref | 16.0 | Ref | 171 | Ref |
| Yes | | 6812 | 4.17 | 0.0027 | 6.68 | <0.0001 | 0.79 | <0.0001 | 16.0 | 0.8928 | 172 | 0.5284 |
| Alcohol consumption during pregnancy No | | 17255 | 4.20 | Ref | 6.44 | Ref | 0.75 | Ref | 16.0 | Ref | 171 | Ref |
| Yes | | 578 | 4.24 | 0.0991 | 7.35 | <0.0001 | 0.82 | 0.0014 | 15.8 | 0.1402 | 174 | 0.0012 |
| Primipara Yes | | 6927 | 4.08 | Ref | 6.51 | Ref | 0.75 | Ref | 15.8 | Ref | 168 | Ref |
| No | | 10596 | 4.28 | <0.0001 | 6.44 | 0.0029 | 0.76 | 0.0001 | 16.1 | <0.0001 | 173 | <0.0001 |
| Study area (Regional Centre) Hokkaido | | 1384 | 3.24 | Ref | 5.84 | Ref | 0.72 | Ref | 15.5 | Ref | 165 | Ref |
| Miyagi | | 1572 | 4.61 | <0.0001 | 6.67 | <0.0001 | 0.85 | <0.0001 | 17.1 | <0.0001 | 172 | <0.0001 |
| Fukushima | | 1968 | 3.93 | <0.0001 | 6.32 | <0.0001 | 0.78 | <0.0001 | 17.1 | <0.0001 | 172 | <0.0001 |
| Chiba | | 1062 | 5.09 | <0.0001 | 5.67 | 0.9352 | 0.69 | 0.3487 | 15.7 | 0.9904 | 174 | <0.0001 |
| Kanagawa | | 1083 | 4.84 | <0.0001 | 5.75 | 0.2521 | 0.73 | 0.7439 | 16.1 | 0.0262 | 173 | <0.0001 |
| Koshin | | 1379 | 4.82 | <0.0001 | 6.63 | <0.0001 | 0.71 | 1.0000 | 15.6 | 1.0000 | 173 | <0.0001 |
| Toyama | | 1026 | 4.25 | <0.0001 | 7.33 | <0.0001 | 1.04 | <0.0001 | 15.5 | 0.9994 | 172 | <0.0001 |
| Aichi | | 1074 | 4.33 | <0.0001 | 6.60 | <0.0001 | 0.77 | 0.0009 | 16.6 | <0.0001 | 176 | <0.0001 |
| Kyoto | | 659 | 3.85 | <0.0001 | 6.78 | <0.0001 | 0.80 | 0.0008 | 15.4 | 0.9963 | 169 | <0.0001 |
| Osaka | | 1513 | 3.93 | <0.0001 | 6.49 | <0.0001 | 0.75 | 0.2386 | 15.5 | 1.0000 | 172 | <0.0001 |
| Hyogo | | 967 | 3.70 | <0.0001 | 6.54 | <0.0001 | 0.73 | 0.9092 | 15.8 | 0.9841 | 172 | <0.0001 |
| Tottori | | 586 | 3.72 | <0.0001 | 6.99 | <0.0001 | 0.75 | 0.9696 | 14.9 | 0.1087 | 169 | <0.0001 |
| Kochi | | 1145 | 5.59 | <0.0001 | 6.65 | <0.0001 | 0.68 | 0.0191 | 15.7 | 0.8252 | 177 | <0.0001 |
| Fukuoka | | 1390 | 3.33 | 0.5346 | 6.38 | <0.0001 | 0.69 | 0.2102 | 16.0 | 0.0432 | 167 | 0.0034 |
| South Kyushu/Okinawa | | 1189 | 3.84 | <0.0001 | 6.98 | <0.0001 | 0.65 | <0.0001 | 15.3 | 0.4273 | 167 | 0.0928 |
| Food consumption (quartile or categorical) | |  |  |  |  |  |  |  |  |  |  |  |
| Grain (g day^-1^) | <354.2 | 3691 | 4.02 | Ref | 6.55 | Ref | 0.73 | Ref | 15.7 | Ref | 171 | Ref |
|  | <438.7 | 3797 | 4.18 | 0.0001 | 6.45 | 0.0142 | 0.74 | 0.0457 | 15.9 | 0.4593 | 172 | 0.0022 |
|  | <528 | 3869 | 4.28 | <0.0001 | 6.39 | 0.0092 | 0.76 | <0.0001 | 16.1 | 0.0005 | 171 | 0.8328 |
|  | 528≤ | 3868 | 4.35 | <0.0001 | 6.52 | 0.6612 | 0.79 | <0.0001 | 16.2 | <0.0001 | 172 | <0.0001 |
| Tubers/starch (g day^-1^) | <10.7 | 3622 | 4.12 | Ref | 6.55 | Ref | 0.76 | Ref | 16.0 | Ref | 172 | Ref |
|  | <19.3 | 3922 | 4.23 | 0.0717 | 6.47 | 0.0432 | 0.74 | 0.6171 | 15.9 | 0.6447 | 172 | 0.8998 |
|  | <31.0 | 3845 | 4.16 | 0.2277 | 6.47 | 0.0249 | 0.76 | 0.5446 | 16.0 | 0.9631 | 172 | 0.5900 |
|  | 31.0≤ | 3836 | 4.32 | 0.0003 | 6.42 | 0.0376 | 0.76 | 0.3171 | 16.0 | 0.9683 | 170 | 0.0010 |
| Sugar/sweets (taken during pregnancy) | No | 9515 | 4.23 | Ref | 6.45 | Ref | 0.76 | Ref | 16.0 | Ref | 172 | Ref |
|  | Yes | 5710 | 4.18 | 0.5662 | 6.51 | 0.4438 | 0.74 | 0.0176 | 15.9 | 0.2429 | 171 | 0.0006 |
| Beans (g day^-1^) | <17.2 | 3630 | 3.95 | Ref | 6.50 | Ref | 0.74 | Ref | 16.0 | Ref | 172 | Ref |
|  | <34.0 | 3892 | 4.26 | <0.0001 | 6.45 | 0.1535 | 0.75 | 0.3656 | 16.0 | 1.0000 | 172 | 0.9192 |
|  | <64.0 | 3859 | 4.28 | <0.0001 | 6.47 | 0.9670 | 0.76 | 0.0010 | 16.0 | 0.9994 | 172 | 0.7871 |
|  | 64.0≤ | 3844 | 4.33 | <0.0001 | 6.49 | 0.8695 | 0.76 | 0.0091 | 15.8 | 0.0962 | 170 | <0.0001 |
| Nuts (taken during pregnancy) | No | 8660 | 4.16 | Ref | 6.44 | Ref | 0.75 | Ref | 16.0 | Ref | 172 | Ref |
|  | Yes | 6565 | 4.27 | 0.0004 | 6.52 | 0.9717 | 0.76 | 0.0004 | 15.9 | 0.2728 | 171 | <0.0001 |
| Vegetables (g day^-1^) | <95.4 | 3686 | 4.02 | Ref | 6.47 | Ref | 0.75 | Ref | 16.0 | Ref | 172 | Ref |
|  | <149.2 | 3812 | 4.11 | 0.1514 | 6.41 | 0.0586 | 0.75 | 0.3705 | 16.1 | 0.9701 | 172 | 0.5355 |
|  | <223.3 | 3914 | 4.28 | <0.0001 | 6.44 | 0.2298 | 0.75 | 0.2427 | 15.9 | 0.8485 | 171 | 0.2887 |
|  | 223.3≤ | 3813 | 4.43 | <0.0001 | 6.59 | 0.0639 | 0.76 | 0.0612 | 16.0 | 0.2704 | 171 | 0.0160 |
| Fruits (g day^-1^) | <48.8 | 3693 | 4.08 | Ref | 6.51 | Ref | 0.76 | Ref | 15.9 | Ref | 172 | Ref |
|  | <109.3 | 3832 | 4.24 | 0.0083 | 6.48 | 0.1697 | 0.76 | 0.9410 | 15.9 | 0.9318 | 172 | 0.6009 |
|  | <193.4 | 3861 | 4.20 | 0.0024 | 6.48 | 0.0723 | 0.75 | 0.9800 | 16.0 | 0.2427 | 171 | 0.0248 |
|  | 193.4≤ | 3839 | 4.31 | <0.0001 | 6.44 | 0.2334 | 0.74 | 0.3144 | 16.1 | 0.1470 | 171 | 0.0107 |
| Mushrooms (g day^-1^) | <2.7 | 2777 | 3.88 | Ref | 6.41 | Ref | 0.74 | Ref | 16.3 | Ref | 171 | Ref |
|  | <6.4 | 4471 | 4.17 | <0.0001 | 6.42 | 0.9145 | 0.74 | 0.6084 | 16.0 | 0.0493 | 171 | 0.8666 |
|  | <12.9 | 3717 | 4.32 | <0.0001 | 6.52 | 0.9233 | 0.76 | 0.0448 | 15.9 | 0.0011 | 172 | 0.2470 |
|  | 12.9≤ | 4260 | 4.38 | <0.0001 | 6.54 | 0.0895 | 0.77 | <0.0001 | 15.9 | 0.0009 | 171 | 0.9979 |
| Seaweeds (g day^-1^) | <1.5 | 3152 | 3.99 | Ref | 6.39 | Ref | 0.74 | Ref | 16.1 | Ref | 172 | Ref |
|  | <4.3 | 4165 | 4.19 | <0.0001 | 6.47 | 0.6053 | 0.75 | 0.0743 | 15.9 | 0.6406 | 172 | 0.9927 |
|  | <8.3 | 4011 | 4.26 | <0.0001 | 6.49 | 0.0805 | 0.75 | 0.0012 | 16.0 | 0.9298 | 172 | 0.9608 |
|  | 8.3≤ | 3897 | 4.36 | <0.0001 | 6.53 | 0.0280 | 0.78 | <0.0001 | 16.0 | 0.9972 | 171 | 0.0740 |
| Seafood (g day^-1^) | <14.3 | 3655 | 3.60 | Ref | 6.43 | Ref | 0.74 | Ref | 16.0 | Ref | 170 | Ref |
|  | <28.0 | 3754 | 3.94 | <0.0001 | 6.44 | 0.4505 | 0.75 | 0.4123 | 16.1 | 0.1868 | 170 | 0.7762 |
|  | <46.0 | 3946 | 4.37 | <0.0001 | 6.49 | 0.5431 | 0.76 | 0.0071 | 15.9 | 0.8938 | 172 | 0.0009 |
|  | 46.0≤ | 3870 | 4.88 | <0.0001 | 6.54 | 0.5522 | 0.77 | 0.0002 | 15.9 | 0.6039 | 174 | <0.0001 |
| Meat (g day^-1^) | <37.7 | 3644 | 4.16 | Ref | 6.47 | Ref | 0.77 | Ref | 16.0 | Ref | 172 | Ref |
|  | <59.0 | 3875 | 4.24 | 0.1379 | 6.46 | 0.8876 | 0.75 | 0.0194 | 15.9 | 0.7573 | 172 | 0.3489 |
|  | <89.7 | 3843 | 4.20 | 0.3447 | 6.49 | 0.9676 | 0.74 | 0.0025 | 15.9 | 0.9788 | 171 | 0.1277 |
|  | 89.7≤ | 3863 | 4.24 | 0.1681 | 6.50 | 0.8838 | 0.75 | 0.0013 | 16.1 | 0.9906 | 171 | 0.0380 |
| Eggs (g day^-1^) | <10.7 | 1569 | 4.16 | Ref | 6.68 | Ref | 0.77 | Ref | 16.0 | Ref | 173 | Ref |
|  | <25.0 | 4281 | 4.21 | 0.8935 | 6.54 | 0.3814 | 0.76 | 0.6078 | 15.9 | 0.8439 | 172 | 0.3507 |
|  | <39.3 | 5054 | 4.18 | 0.6954 | 6.41 | 0.0036 | 0.75 | 0.3624 | 16.0 | 0.9784 | 172 | 0.1820 |
|  | 39.3≤ | 4321 | 4.26 | 0.1680 | 6.43 | 0.0193 | 0.74 | 0.2168 | 16.0 | 0.9531 | 170 | <0.0001 |
| Dairy (g day^-1^) | <104.2 | 3705 | 4.20 | Ref | 6.69 | Ref | 0.78 | Ref | 15.9 | Ref | 173 | Ref |
|  | <200 | 3847 | 4.21 | 0.9576 | 6.56 | 0.0021 | 0.76 | 0.3886 | 16.0 | 1.0000 | 172 | 0.0732 |
|  | <325 | 3830 | 4.21 | 0.6408 | 6.37 | <0.0001 | 0.75 | 0.0416 | 16.0 | 0.8276 | 171 | <0.0001 |
|  | 325≤ | 3843 | 4.22 | 0.8061 | 6.29 | <0.0001 | 0.73 | 0.0010 | 16.0 | 0.9785 | 169 | <0.0001 |
| Fat (g day^-1^) | <6.6 | 3573 | 4.10 | Ref | 6.48 | Ref | 0.77 | Ref | 16.0 | Ref | 171 | Ref |
|  | <9.7 | 3873 | 4.21 | 0.0126 | 6.45 | 0.4110 | 0.75 | 0.9241 | 15.9 | 0.4576 | 170 | 0.2335 |
|  | <13.7 | 3867 | 4.24 | 0.0094 | 6.47 | 0.6084 | 0.75 | 0.3900 | 16.0 | 0.9158 | 172 | 0.0628 |
|  | 13.7≤ | 3912 | 4.29 | <0.0001 | 6.51 | 0.8780 | 0.76 | 0.2763 | 16.0 | 0.9998 | 173 | <0.0001 |
| Confectionery (g day^-1^) | <9.3 | 3623 | 4.40 | Ref | 6.56 | Ref | 0.77 | Ref | 16.0 | Ref | 173 | Ref |
|  | <18.4 | 3793 | 4.26 | 0.1046 | 6.46 | 0.0369 | 0.75 | 0.3266 | 15.9 | 0.7199 | 172 | 0.1292 |
|  | <31.7 | 3911 | 4.14 | <0.0001 | 6.45 | 0.0007 | 0.74 | 0.3340 | 16.0 | 1.0000 | 171 | <0.0001 |
|  | 31.7≤ | 3898 | 4.05 | <0.0001 | 6.45 | 0.0453 | 0.76 | 0.8206 | 16.0 | 0.9611 | 170 | <0.0001 |
| Beverages (ml day^-1^), non-alcoholic | <125.7 | 3470 | 4.13 | Ref | 6.21 | Ref | 0.73 | Ref | 15.9 | Ref | 170 | Ref |
|  | <270.7 | 4032 | 4.19 | 0.7707 | 6.33 | 0.0003 | 0.74 | 0.1013 | 16.0 | 0.6565 | 172 | 0.0017 |
|  | <477.9 | 3857 | 4.19 | 0.6658 | 6.52 | <0.0001 | 0.76 | 0.0038 | 15.9 | 0.8459 | 171 | 0.0050 |
|  | 477.9≤ | 3866 | 4.32 | 0.0606 | 6.82 | <0.0001 | 0.78 | <0.0001 | 16.1 | 0.0948 | 173 | <0.0001 |
| Seasoning/spices (g day^-1^) | <9.7 | 3675 | 4.05 | Ref | 6.51 | Ref | 0.75 | Ref | 16.0 | Ref | 172 | Ref |
|  | <15.0 | 3844 | 4.15 | 0.1139 | 6.45 | 0.4423 | 0.73 | 0.9563 | 16.0 | 0.9868 | 172 | 0.4642 |
|  | <22.0 | 3883 | 4.22 | 0.0006 | 6.42 | 0.4746 | 0.75 | 0.1821 | 15.9 | 0.2448 | 171 | 0.9190 |
|  | 22.0≤ | 3823 | 4.42 | <0.0001 | 6.52 | 0.6452 | 0.79 | <0.0001 | 16.0 | 0.9630 | 171 | 0.1794 |
| Tap/well water (times per week) | <1 | 10007 | 4.16 | Ref | 6.39 | Ref | 0.75 | Ref | 15.9 | Ref | 172 | Ref |
|  | 1–2 | 1332 | 4.30 | 0.1784 | 6.49 | 0.0279 | 0.75 | 0.9577 | 15.9 | 0.8789 | 171 | 0.0975 |
|  | 3≤ | 3886 | 4.31 | 0.0112 | 6.69 | <0.0001 | 0.77 | 0.0053 | 16.1 | 0.1206 | 171 | 0.0038 |
| Bottled/filtered water (times per week) | <1 | 7061 | 4.21 | Ref | 6.58 | Ref | 0.77 | Ref | 16.0 | Ref | 172 | Ref |
|  | 1–2 | 1764 | 4.18 | 0.8607 | 6.47 | 0.0918 | 0.76 | 0.6205 | 16.0 | 0.5778 | 172 | 0.9281 |
|  | 3≤ | 6400 | 4.21 | 0.9189 | 6.36 | <0.0001 | 0.74 | 0.0043 | 16.0 | 0.8005 | 171 | 0.4945 |

*Abbreviations:* Hg, mercury; Pb, lead; Cd, cadmium; Mn, manganese; Se, selenium.

Steel's test was performed for nonparametric multiple comparison.
