# Supplementary material for: Blood mercury, lead, cadmium, manganese and selenium levels in pregnant women and their determinants: the Japan Environment and Children’s Study (JECS)
Source: J Expo Sci Environ Epidemiol. 2019 Apr 18;29(5):633–47. doi: 10.1038/s41370-019-0139-0 (PMC6760604; doi:10.1038/s41370-019-0139-0)
Supplement: Supplementary file 8 — Supplementary TableS6 [file 41370_2019_139_MOESM8_ESM.docx]

Table S6. Associations between blood metal concentrations and occupational exposure

|  |  | Hg |  | Pb |  | Cd |  | Mn |  | Se |  |
| --- | --- | --- | --- | --- | --- | --- | --- | --- | --- | --- | --- |
|  | *N* | *Mean* | *P* | *Mean* | *P* | *Mean* | *P* | *Mean* | *P* | *Mean* | *P* |
| Kerosene, petroleum, benzene, gasoline |  |  |  |  |  |  |  |  |  |  |  |
| No | 14,191 | 4.20 | Ref | 6.43 | Ref | 0.75 | Ref | 15.9 | Ref | 171 | Ref |
| Yes | 2,858 | 4.19 | 0.1263 | 6.59 | <0.0001 | 0.77 | 0.0842 | 16.1 | 0.3545 | 171 | 0.6104 |
| Chlorine bleach, germicide |  |  |  |  |  |  |  |  |  |  |  |
| No | 12,585 | 4.20 | Ref | 6.48 | Ref | 0.75 | Ref | 16.0 | Ref | 171 | Ref |
| Yes | 4,495 | 4.20 | 0.5330 | 6.40 | 0.0831 | 0.76 | 0.0668 | 16.0 | 0.6695 | 171 | 0.6410 |
| Medical disinfectant |  |  |  |  |  |  |  |  |  |  | . |
| No | 14,959 | 4.19 | Ref | 6.45 | Ref | 0.75 | Ref | 16.0 | Ref | 171 | Ref |
| Yes | 2,093 | 4.23 | 0.1084 | 6.52 | 0.0713 | 0.75 | 0.4092 | 15.9 | 0.6150 | 171 | 0.0752 |
| Permanent marker |  |  |  |  |  |  |  |  |  |  |  |
| No | 10,556 | 4.20 | Ref | 6.47 | Ref | 0.75 | Ref | 16.0 | Ref | 171 | Ref |
| Yes | 6,534 | 4.21 | 0.7743 | 6.45 | 0.7531 | 0.75 | 0.5905 | 15.9 | 0.6935 | 171 | 0.1020 |
| Water-based paint or inkjet printer |  |  |  |  |  |  |  |  |  |  |  |
| No | 12,564 | 4.18 | Ref | 6.46 | Ref | 0.75 | Ref | 16.0 | Ref | 171 | Ref |
| Yes | 4,422 | 4.25 | 0.0227 | 6.46 | 0.0738 | 0.75 | 0.3506 | 15.9 | 0.1951 | 171 | 0.5223 |
| Organic solvents (e.g., paint thinner, solvent for examination/analysis/extraction, dry-cleaning detergent, stain-removing agent, paint coating, and nail polish remover) |  |  |  |  |  |  |  |  |  |  |  |
| No | 15,492 | 4.21 | Ref | 6.46 | Ref | 0.76 | Ref | 16.0 | Ref | 171 | Ref |
| Yes | 1,522 | 4.09 | 0.0168 | 6.51 | 0.4995 | 0.73 | 0.0052 | 15.8 | 0.1378 | 170 | 0.0379 |
| Photocopier, laser printer |  |  |  |  |  |  |  |  |  |  |  |
| No | 11,997 | 4.18 | Ref | 6.44 | Ref | 0.75 | Ref | 16.0 | Ref | 172 | Ref |
| Yes | 5,134 | 4.25 | 0.0016 | 6.51 | 0.0021 | 0.76 | 0.3148 | 15.9 | 0.0685 | 170 | <0.0001 |
| Engine oil |  |  |  |  |  |  |  |  |  |  |  |
| No | 16,883 | 4.20 | Ref | 6.46 | Ref | 0.75 | Ref | 16.0 | Ref | 171 | Ref |
| Yes | 146 | 4.20 | 0.6212 | 6.64 | 0.4117 | 0.74 | 0.4053 | 15.8 | 0.7086 | 172 | 0.8321 |
| Formalin, formaldehyde |  |  |  |  |  |  |  |  |  |  |  |
| No | 16,934 | 4.20 | Ref | 6.46 | Ref | 0.75 | Ref | 16.0 | Ref | 171 | Ref |
| Yes | 104 | 4.63 | 0.0814 | 6.73 | 0.0680 | 0.73 | 0.1653 | 15.7 | 0.3508 | 173 | 0.4215 |
| Anticancer drug (excluding those prescribed to the respondent) |  |  |  |  |  |  |  |  |  |  |  |
| No | 16,842 | 4.20 | Ref | 6.46 | Ref | 0.75 | Ref | 16.0 | Ref | 171 | Ref |
| Yes | 175 | 4.40 | 0.0957 | 6.67 | 0.3225 | 0.69 | 0.0374 | 16.0 | 0.9420 | 171 | 0.8575 |
| General anesthetic for surgery |  |  |  |  |  |  |  |  |  |  |  |
| No | 16,902 | 4.20 | Ref | 6.46 | Ref | 0.75 | Ref | 16.0 | Ref | 171 | Ref |
| Yes | 127 | 4.53 | 0.0300 | 6.91 | 0.1181 | 0.74 | 0.7659 | 16.0 | 0.9281 | 170 | 0.4219 |
| Insecticide |  |  |  |  |  |  |  |  |  |  |  |
| No | 15,520 | 4.19 | Ref | 6.46 | Ref | 0.75 | Ref | 16.0 | Ref | 171 | Ref |
| Yes | 1,504 | 4.26 | 0.6931 | 6.47 | 0.2484 | 0.76 | 0.3769 | 16.0 | 0.9177 | 170 | 0.0557 |
| Herbicide |  |  |  |  |  |  |  |  |  |  |  |
| No | 16,671 | 4.20 | Ref | 6.46 | Ref | 0.75 | Ref | 16.0 | Ref | 171 | Ref |
| Yes | 186 | 4.10 | 0.1253 | 6.72 | 0.5266 | 0.80 | 0.0871 | 16.2 | 0.4624 | 169 | 0.1107 |
| Radiation, radioactive substances, isotopes |  |  |  |  |  |  |  |  |  |  |  |
| No | 16,760 | 4.20 | Ref | 6.46 | Ref | 0.75 | Ref | 16.0 | Ref | 171 | Ref |
| Yes | 242 | 4.34 | 0.1142 | 6.58 | 0.5496 | 0.74 | 0.7603 | 15.4 | 0.0607 | 171 | 0.8787 |
| Microbes |  |  |  |  |  |  |  |  |  |  |  |
| No | 16,972 | 4.20 | Ref | 6.46 | Ref | 0.75 | Ref | 16.0 | Ref | 171 | ref |
| Yes | 52 | 3.94 | 0.7425 | 6.93 | 0.7421 | 0.73 | 0.6460 | 15.7 | 0.5685 | 170 | 0.5085 |
| Any products containing lead (e.g., solder) |  |  |  |  |  |  |  |  |  |  |  |
| No | 16,966 | 4.20 | Ref | 6.46 | Ref | 0.75 | Ref | 16.0 | Ref | 171 | Ref |
| Yes | 54 | 3.75 | 0.2402 | 7.24 | 0.2570 | 0.72 | 0.4488 | 15.8 | 0.8525 | 172 | 0.6881 |
| Lead-free solder |  |  |  |  |  |  |  |  |  |  |  |
| No | 16,973 | 4.20 | Ref | 6.46 | Ref | 0.75 | Ref | 16.0 | Ref | 171 | Ref |
| Yes | 42 | 3.99 | 0.9559 | 6.55 | 0.9587 | 0.80 | 0.9042 | 16.4 | 0.5483 | 170 | 0.8965 |
| Chromium, arsenic, cadmium |  |  |  |  |  |  |  |  |  |  |  |
| No | 17,002 | 4.20 | Ref | 6.46 | Ref | 0.75 | Ref | 16.0 | Ref | 171 | Ref |
| Yes | 6 | 3.91 | 0.7792 | 5.04 | 0.0999 | 0.80 | 0.4493 | 15.3 | 0.2789 | 167 | 0.7365 |
| Mercury |  |  |  |  |  |  |  |  |  |  |  |
| No | 16,880 | 4.20 | Ref | 6.46 | Ref | 0.75 | Ref | 16.0 | Ref | 171 | Ref |
| Yes | 121 | 3.84 | 0.2979 | 6.52 | 0.5860 | 0.75 | 0.8424 | 16.3 | 0.8746 | 167 | 0.0908 |
| Dyestuffs (for hair coloring) |  |  |  |  |  |  |  |  |  |  |  |
| No | 15,065 | 4.23 | Ref | 6.45 | Ref | 0.75 | Ref | 16.0 | Ref | 171 | Ref |
| Yes | 1,671 | 3.98 | <0.0001 | 6.53 | 0.0795 | 0.76 | 0.4353 | 15.9 | 0.4871 | 170 | 0.0222 |

*Abbreviations:* Hg, mercury; Pb, lead; Cd, cadmium; Mn, manganese; Se, selenium.

The Wilcoxon nonparametric test was performed.
