# Supplementary figures and images for: Blood mercury, lead, cadmium, manganese and selenium levels in pregnant women and their determinants: the Japan Environment and Children’s Study (JECS)
Source: J Expo Sci Environ Epidemiol. 2019 Apr 18;29(5):633–47. doi: 10.1038/s41370-019-0139-0 (PMC6760604; doi:10.1038/s41370-019-0139-0)

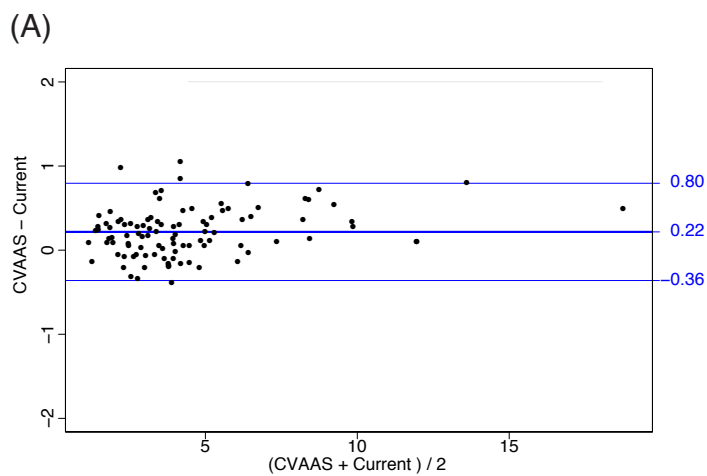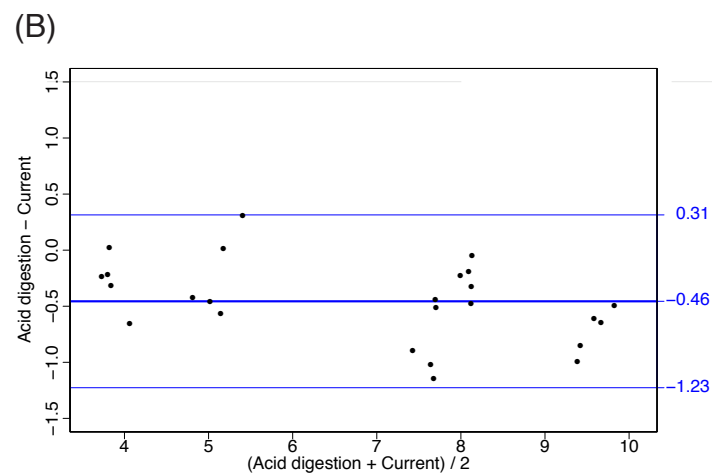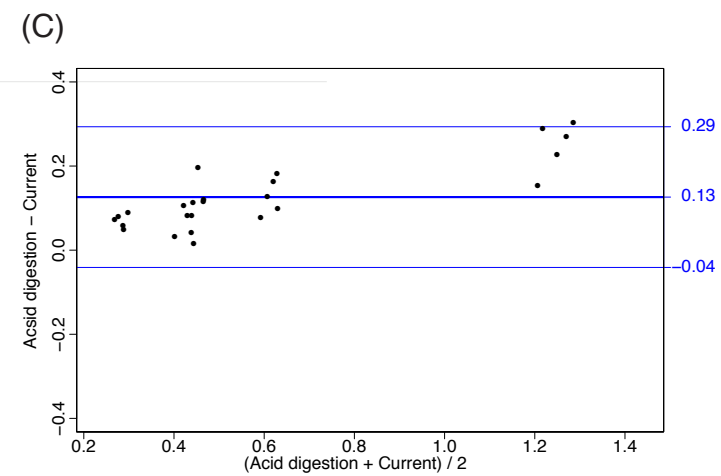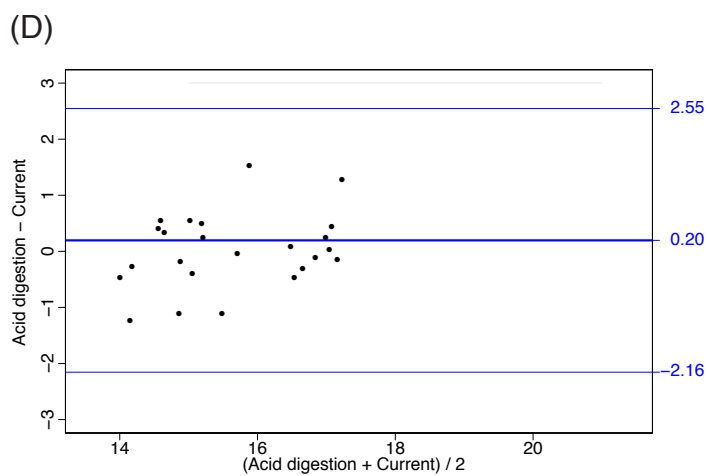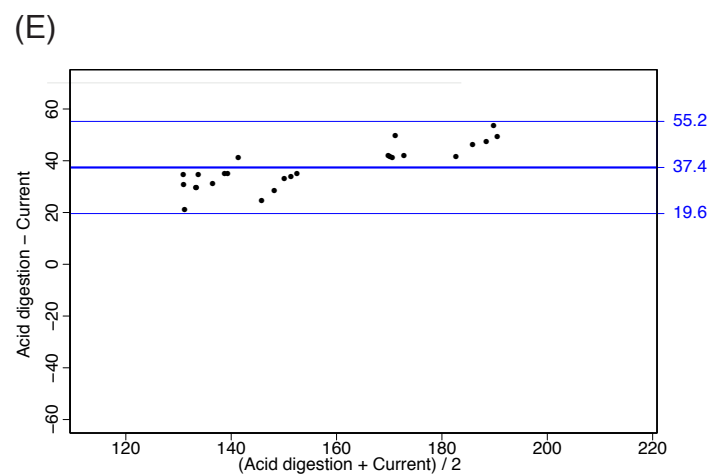

Figure S1

Supplement: Supplementary file 9 — Supplementary FigureS1 [file 41370_2019_139_MOESM9_ESM.pdf]

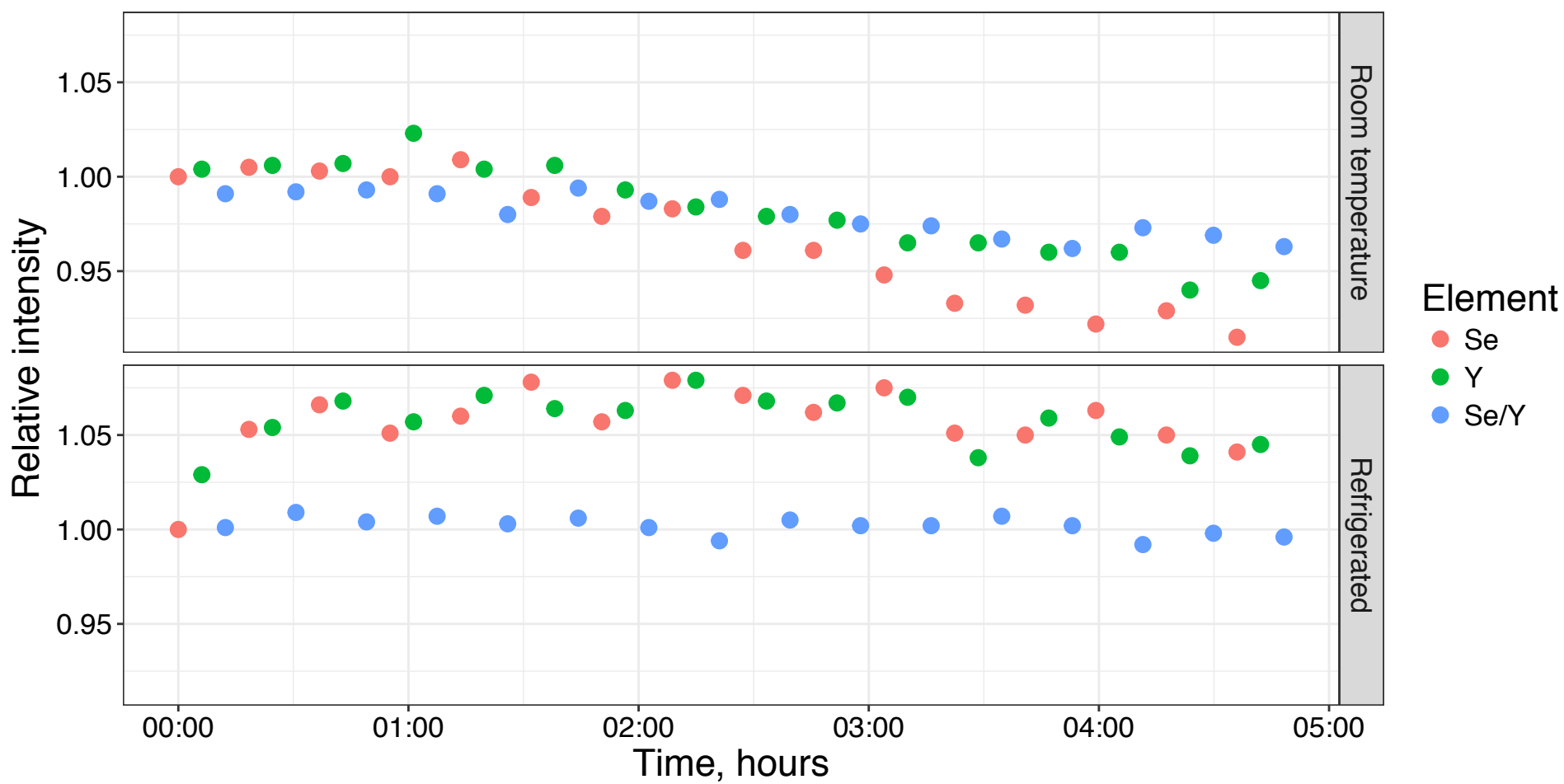

Figure S2

Supplement: Supplementary file 10 — Supplementary FigureS2 [file 41370_2019_139_MOESM10_ESM.pdf]

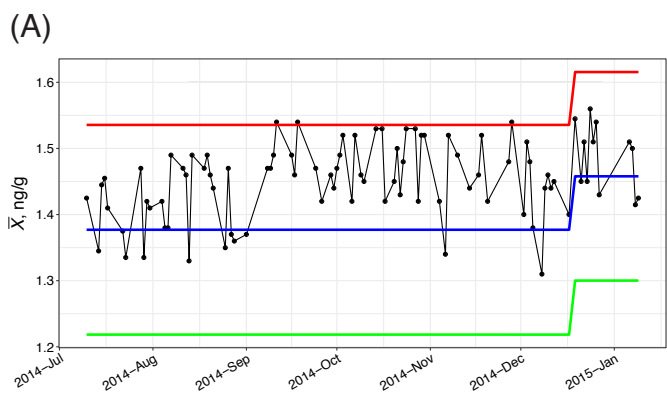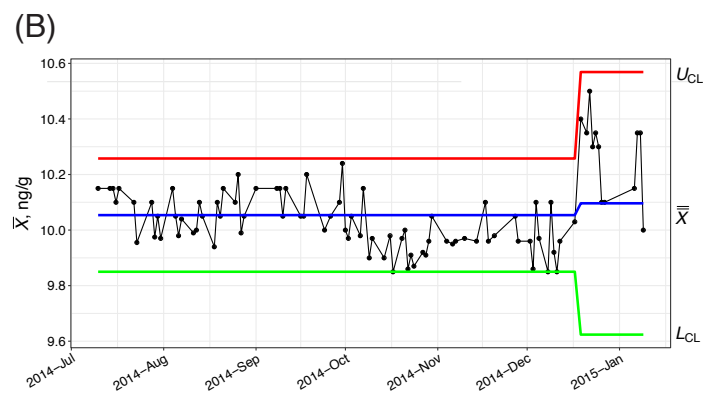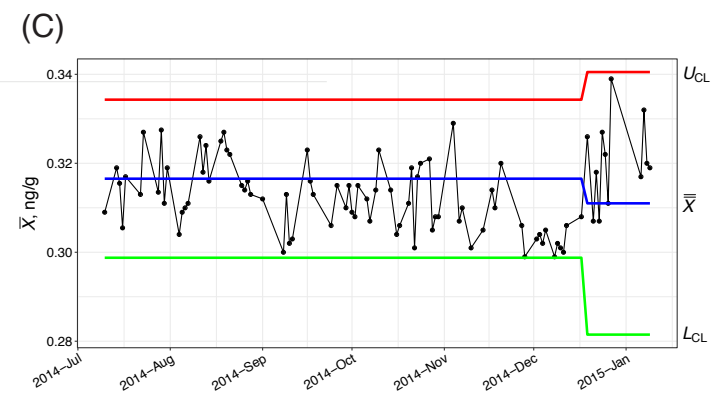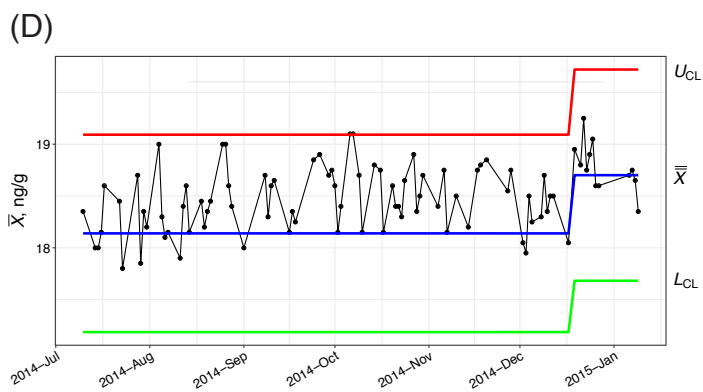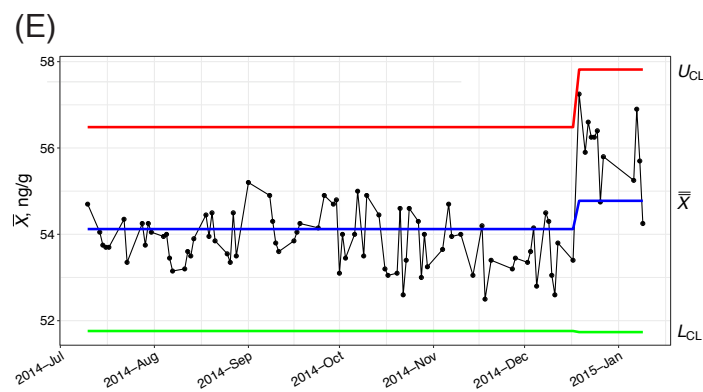

Figure S3

Supplement: Supplementary file 11 — Supplementary FigureS3 [file 41370_2019_139_MOESM11_ESM.pdf]

(A)

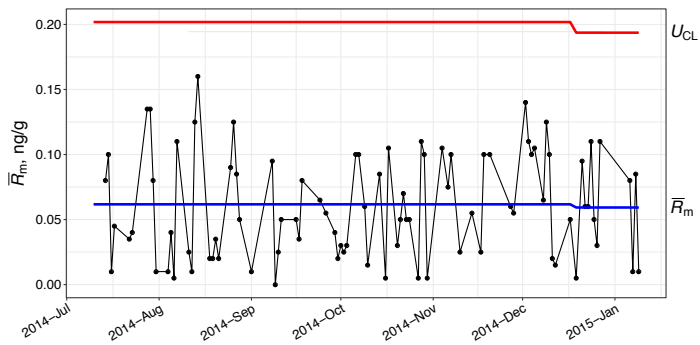

(B)

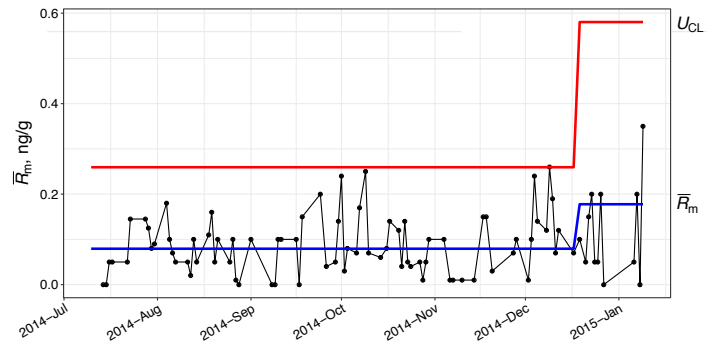

(C)

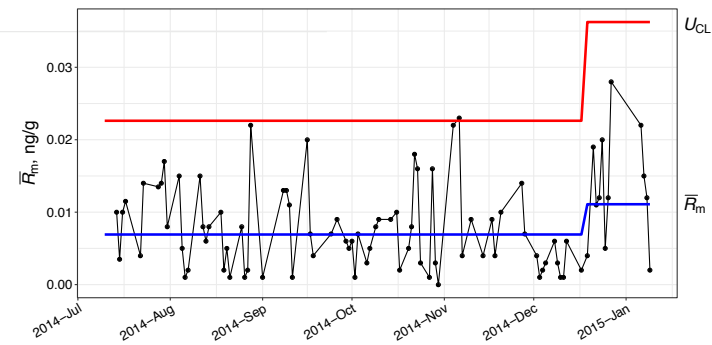

(D)

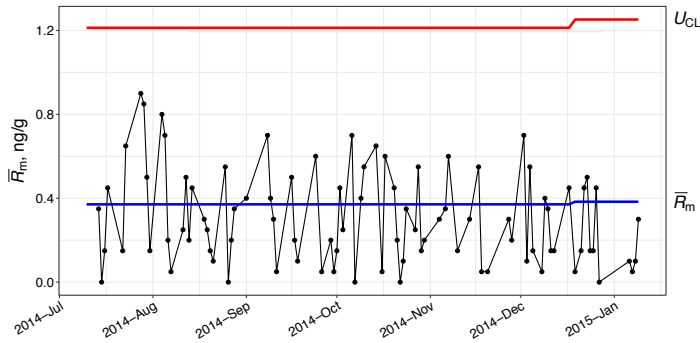

(E)

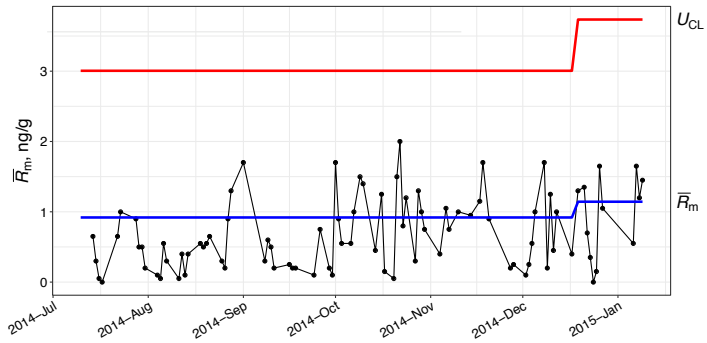

Figure S4

Supplement: Supplementary file 12 — Supplementary FigureS4 [file 41370_2019_139_MOESM12_ESM.pdf]

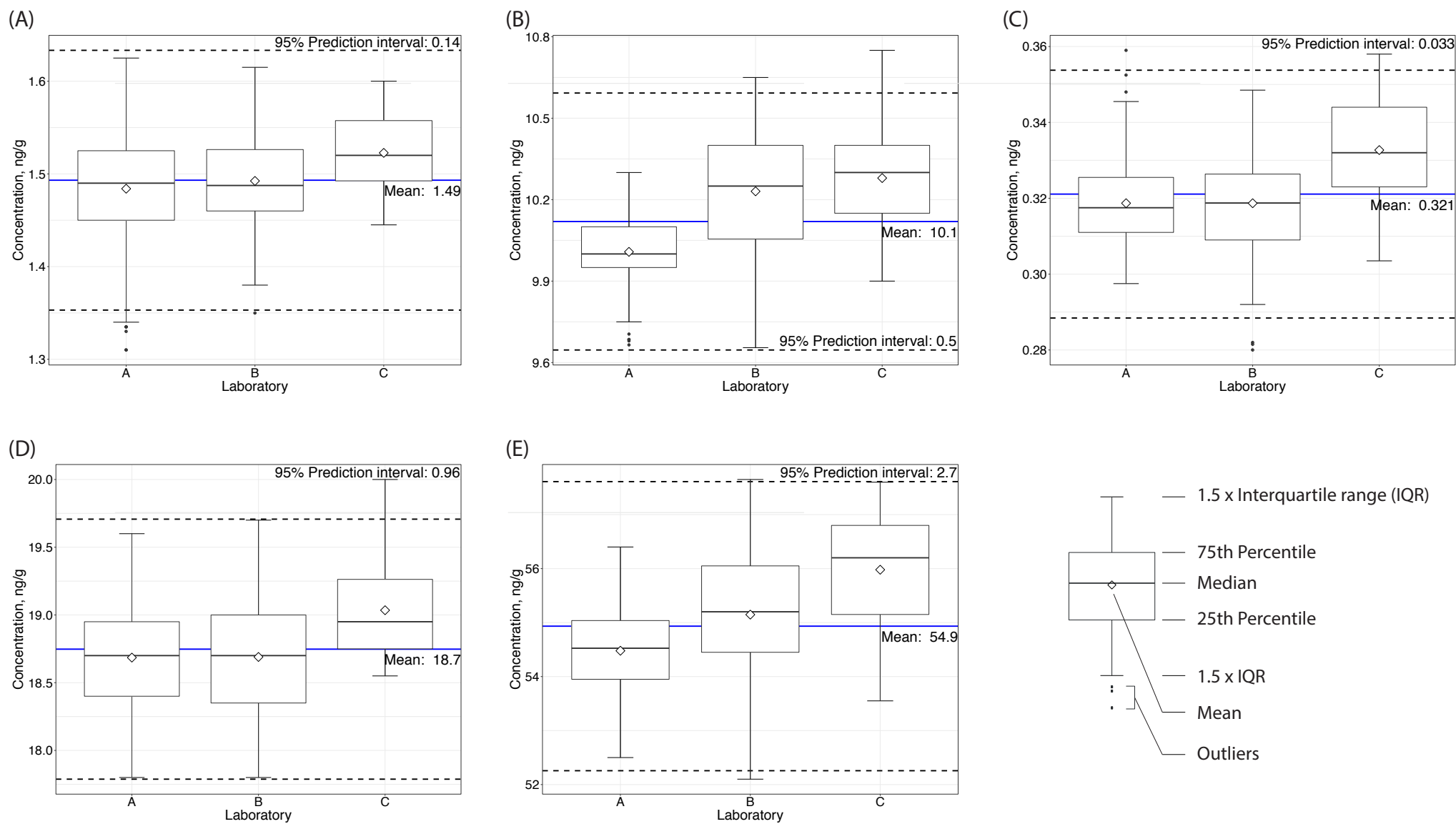

Figure S5

Supplement: Supplementary file 13 — Supplementary FigureS5 [file 41370_2019_139_MOESM13_ESM.pdf]
